# Identification of serum protein biomarkers for pre‐cancerous lesions associated with pancreatic ductal adenocarcinoma

**DOI:** 10.1002/1878-0261.70213

**Published:** 2026-02-18

**Authors:** Hannah Mearns, Jaclyn S. Long, Sergio Lilla, Kelly Hodge, Marcus J. G. W. Ladds, Colin Nixon, Paula Fernández‐Palanca, Sara Zanivan, Pilar Acedo, Stephen P. Pereira, Kevin M. Ryan

**Affiliations:** ^1^ Cancer Research UK Scotland Institute Glasgow UK; ^2^ School of Cancer Sciences University of Glasgow UK; ^3^ Institute for Liver and Digestive Health, Royal Free Hospital Campus, University College London (UCL) UK; ^4^ Present address: AstraZeneca Cambridge UK; ^5^ Present address: Department of Experimental Therapeutics MD Anderson Cancer Center Houston TX USA

**Keywords:** autophagy, biomarker, early detection, mice, pancreatic ductal adenocarcinoma; pancreatic intraepithelial neoplasia, proteomics, serum

## Abstract

Pancreatic ductal adenocarcinoma (PDAC) has poor prognosis as early‐stage asymptomaticity leads to late‐stage diagnoses. Strategies to detect PDAC earlier or identify high‐risk individuals are therefore paramount. Here, we report results from genetically engineered mice and PDAC patients that identify serum proteins associated with pancreatic intraepithelial neoplasms (PanINs), the most common PDAC precursor, and early‐stage PDAC. Initially, we screened previously described PanIN‐abundant mice, harbouring pancreatic and duodenal homeobox 1 (*Pdx1*)*‐Cre*, Lox‐STOP‐Lox‐*Kras* ^G12D/+^ and floxed alleles of essential autophagy genes autophagy‐related 7 (*Atg7*) or autophagy‐related 5 (*Atg5*). Sera from these mice were assessed by proteomics and hits were compared to those in Lox‐STOP‐Lox‐*Kras* ^G12D/+^ Lox‐STOP‐Lox‐*Trp53* ^R172H/+^ *Pdx1‐Cre* (KPC) mice, which closely recapitulate human disease, and early‐stage (I–II) PDAC patients. Levels of inter‐alpha‐trypsin inhibitor heavy chain H3 (ITIH3) were significantly elevated in all three screens, with complement C5, complement factors B and H (CFB/CFH), and monocyte differentiation antigen CD14 increased in KPC mice and PDAC patients; and all were significantly increased co‐ordinately in PDAC according to disease stage. Serum levels of C5, CFH and CD14 together constitute a novel panel for identifying PanINs and early‐stage PDAC with confidence, and when combined with additional screening, could help increase survival from this dismal disease.

## Graphical abstract

This work identified serum proteins associated with pancreatic epithelial neoplasms (PanINs) and early‐stage PDAC. Proteomics screens assessed genetically engineered mice with abundant PanINs, KPC mice (Lox‐STOP‐Lox‐*Kras* ^G12D/+^ Lox‐STOP‐Lox‐*Trp53* ^R172H/+^ *Pdx1‐Cre*) before PDAC development and also early‐stage PDAC patients (n = 31), compared to benign pancreaticobiliary disease case controls. A novel panel of serum proteins was found which could identify PanINs and early‐stage PDAC with confidence.

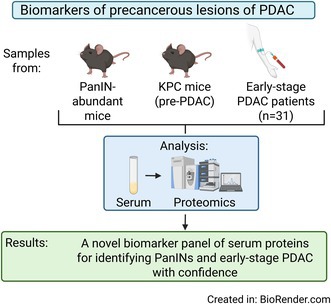

**Abbreviations**
ANOVA: Analysis of variance
ATG: Autophagy‐related
C5: Complement C5
CD14: Monocyte differentiation antigen CD14
CFB: Complement factor B
CFH: Complement factor H
CPTAC: Clinical proteomic tumour analysis consortium
ECM: Extracellular matrix
GEMM: Genetically engineered mouse model
IHC: Immunohistochemistry
ITIH3: Inter‐alpha‐trypsin inhibitor heavy chain H3
KPC: *Kras* ^G12D/+^ *Trp53* ^R172H/+^ *Pdx1‐Cre*
MS: Mass spectrometry
PanIN: Pancreatic intraepithelial neoplasia
PDAC: Pancreatic ductal adenocarcinoma
UALCAN: University of Alabama at Birmingham Cancer data analysis portal

## 1 Introduction

Despite substantial advances in cancer research, there remains a large clinical unmet need for pancreatic cancer patients, around 95% of whom present with pancreatic ductal adenocarcinoma (PDAC). The unmet need arises from the poor prognosis of these patients, with only around 10% surviving 5 years after diagnosis [1]. This is largely due to late diagnoses because of a lack of specific symptoms at early stages of disease. Thus, the focus moves to early detection and in particular, the use of noninvasive biomarkers to indicate the presence of precancerous lesions at a high risk of progressing to PDAC.

Various cancer‐associated biomarkers have been identified and are used by clinicians to aid in cancer detection and diagnosis, such as prostate‐specific antigen (PSA) for prostate cancer [2]. For pancreatic cancer patients, clinicians use serum levels of carbohydrate antigen 19–9 (CA19‐9), a glycan also referred to as sialyl‐Lewis A, to assess disease stage as well as to monitor therapy responses in patients [3, 4]. However, CA19‐9 has proven to be unsuitable for screening asymptomatic patients, as other conditions, aside from pancreatic cancer, can result in elevated CA19‐9 levels, and it is not applicable in Lewis‐antigen‐negative patients, as they lack an α1‐4‐fucosyltransferase called FUT3 and thus cannot synthesise CA19‐9 [5]. As a result, early detection biomarkers for pancreatic cancer require further development.

PDAC can arise from various precancerous lesions, the most common being pancreatic intraepithelial neoplasia (PanINs). These lesions emerge following accumulation of mutations, progress through levels of dysplasia and can advance to PDAC over time [6].

By definition, PanINs are less than 5 mm in diameter and so are hard to identify on noninvasive imaging such as computerised tomography (CT), making it very challenging to obtain samples from patients with PanINs but no PDAC. Detectable changes in the serum proteome resulting from precancerous lesions or early‐stage cancer have been associated with several cancer types, including gastric cancer, ovarian cancer and oropharyngeal cancer amongst many others [7, 8, 9]. This highlights the use of serum proteomics as a useful tool for unbiased screening for precancerous and early‐stage cancerous changes. However, due to the low abundance of PanINs in patients, identification of specific markers of PanINs is also very difficult with human samples.

In this study, genetically engineered mouse models (GEMMs) were used to allow the serum proteome of PanIN‐bearing organisms to be assessed. Two GEMMs were used: a model with abundant PanINs for initial PanIN marker detection (Lox‐STOP‐Lox‐*Kras* ^G12D/+^ *Atg7* ^−/−^ *Pdx1‐Cre* and Lox‐STOP‐Lox‐*Kras* ^G12D/+^ *Atg5* ^−/−^ *Pdx1‐Cre*) and then the commonly used KPC model of PDAC (but harvested before PDAC development) (Lox‐STOP‐Lox‐*Kras* ^G12D/+^ Lox‐STOP‐Lox‐*Trp53* ^R172H/+^ *Pdx1‐Cre*), which better mimics PanIN levels seen in patients. Serum from these mice was screened using mass spectrometry (MS)‐based proteomics at specific time points. Following the GEMM serum screens, serum from early‐stage PDAC patients and symptomatic patients diagnosed with benign pancreaticobiliary diseases was also compared to the GEMM data, leading to the identification of markers of PanINs and early‐stage PDAC.

## 2 Materials and methods

### 2.1 Animal work

All experiments were performed in accordance with the UK Home Office guidelines and the Animals (Scientific Procedures) Act (1986), under project licences held by Kevin Ryan (P54E3DD25 and PP5779290), that had been approved by the University of Glasgow's Ethical Review process. *Atg7* ^fl/fl^ mice were from M. Komatsu and have been previously described [10]. *Atg5* ^fl/fl^ mice were obtained from RIKEN and have also been previously described [11]. *Pdx1‐Cre*, *Kras* ^G12D/+^ and *Trp53* ^R172H/+^ were previously described [12, 13, 14]. Mice were housed (up to 5 per cage, separated by sex) inside individually ventilated cages, in pathogen‐free facilities, with a 12‐h light/dark cycle and with food and water *ad libitum*, in 20–24 °C and 45–65% humidity. Mice were maintained on a mixed background (C57BL/6, 129Sv1) and genotyping was conducted by Transnetyx Inc (www.transnetyx.com) when mice were weaned at around 4 week.

The GEMM proteomics screens used a minimum of 10 mice per genotype and per sex. This was based on previous power calculations (of a two‐sided hypothesis, a significance level of 5%, a power of 80% and a signal to noise ratio of 1.5) but keeping in mind the principles of the National centre for the replacement, refinement and reduction of animals in research (NC3R) [15]. Randomisation was done according to the mouse genotype and no blinding was applied in the analysis. Samples in the proteomics screen were only excluded if they had a lower protein amount following digestion, deviating from all other samples or if the KPC mice had large macroscopic PDAC upon dissection.

Mice were bred to obtain various genotypes. First, *Pdx1‐Cre Atg7* ^fl/fl^ *Kras* ^G12D/+^ and *Pdx1‐Cre Atg5* ^fl/fl^ *Kras* ^G12D/+^ mice, to obtain cohorts with abundant PanINs. Several control groups were also generated: *Pdx1‐Cre Atg7* ^fl/fl^ and *Pdx1‐Cre Atg5* ^fl/fl^ mice (which exhibit impaired pancreatic autophagy as described previously [16]), as well as *Pdx1‐Cre*‐only mice and wild‐type mice for each ATG colony. Mice were harvested at 90 days (−/+ 10 days) as this is when PanIN levels were already shown to peak in this model [16]. For the serum screen on KPC mice (a commonly used PDAC GEMM), mice were generated to have *Pdx1‐Cre Kras* ^G12D/+^ *Trp53* ^R172H/+^ [17]. KPC mice were harvested at 12 weeks (84 days) (+/− 10 days) based on preliminary data to have the latest time‐point, but minimum PDAC upon dissection. For details on the comparisons made and the statistical tests used, see section ‘perseus (version 1.6.15.0)’. For both GEMM screens, the majority of the wild‐type samples were the same, where sample volume allowed. This was to reduce the number of mice needed.

Following humane culling by carbon dioxide (CO_2_) inhalation at four litres per minute for 4 min, in a CO_2_ chamber and confirmation of death by cervical dislocation, blood was taken directly from the heart. Blood was left to coagulate for a minimum of 30 min, centrifuged at 4 °C for 15 min at 900× ***g*** to obtain serum (the supernatant), which was then stored at −80 °C until needed. Upon dissection, pancreatic tissue was taken for histological analysis, placed directly into 10% neutral buffered formalin for 24 h and then transferred to 70% ethanol.

Fixed tissues were embedded in paraffin wax and cut to 4 μm using a microtome, placed onto glass slides (nonadhesive slides for haematoxylin and eosin (H&E) staining, but adhesive for alcian blue/periodic acid‐Schiff (AB/PAS) staining and antibody staining) and baked for 2 h at 60 °C. H&E staining (RBA‐4201‐00A, CellPath; RRSP34‐F, Atom Scientific) was conducted on a Leica ST5020 autostainer: dewaxed in xylene, moved through graded ethanol (from a greater to a lower alcohol content) to rehydrate the tissue and then washed with tap water. The slides were stained with haematoxylin z for 13 min, washed in tap water and differentiated in 1% acid alcohol solution. Slides were then exposed to a Scott's tap water substitute, washed in tap water and stained with Putts Eosin for 3 min. For AB/PAS staining (RRSP4‐E and RRSP124‐E, Atom Scientific), slides were as above dewaxed in xylene, moved through graded ethanol and then washed in tap water. Slides were then stained for 10 min with an in‐house Alcian blue solution and then rinsed in tap water. Then, slides were put into 0.5% periodic acid for 7 min, washed with water, put into Schiffs reagent for 20 min and then rinsed with tap water. For the last step for both stains (H&E and AB/PAS), sections were rinsed in tap water, dehydrated in graded ethanol (this time moving from a lower to a greater alcohol content) and put into xylene. Tissues were mounted (using DPX mountant (SEA‐1300‐00A, CellPath)) onto slides and imaged using a Zeiss AX10 light microscope, at magnifications listed in the figure legends.

For the staining of α‐SMA, CFB, CFH, ITIH3, Ki67 and CD14, the Leica Bond Rx autostainer was used. Slides to be stained were loaded onto the autostainer and dewaxing of sections took place using Bond dewax solution (AR9222; Leica, Sheffield, UK). Epitope retrieval using Epitope Retrieval solution 2 (AR9640; Leica, ER2) was performed at 100 °C for α‐SMA (for 30 min), and CFB, CFH, ITIH3 and Ki67 (for 20 min). For CD14, antigen retrieval was performed at 100 °C for 40 min with Epitope Retrieval solution 1 (AR9661; Leica, ER1). Subsequently, sections were rinsed in Bond wash buffer (AR9590; Leica) followed by Peroxidase block using the Intense R kit (DS9263; Leica). Slides were rinsed again with Bond wash buffer before incubating with the corresponding primary antibodies diluted in Bond diluent (AR9352; Leica) at a previously optimised dilution (α‐SMA, Cell Signaling #19245, 1/100; CFB, Proteintech 10 170‐1‐AP, 1/500; CFH, Affinity Biologicals DF6889, 1/75; ITIH3, Proteintech 21 247‐1‐AP, 1/50; Ki67, Cell Signaling #12202, 1/1000; CD14, Proteintech 17000‐1‐AP, 1/750) for 40 min at room temperature. The sections were then rinsed with Bond wash buffer before incubation with the Rabbit Envision secondary antibody (Agilent; K4003, Santa Clara, CA, USA) for 30 min at room temperature. Sections were subsequently rinsed with Bond wash buffer before visualisation with DAB and counterstaining with haematoxylin using the Intense R kit (DS9263; Leica). Finally, slides were rinsed in tap water, dehydrated in graded ethanol and put into xylene before coverslipped using DPX mountant.

For vimentin staining, the Agilent Autostainer Link48 was used. After the slides were dewaxed in xylene, rehydrated through graded ethanol and washed with water, antigen retrieval with Target Retrieval Solution—high pH (K8004; Agilent) was performed at 97 °C using an Agilent PT module. Subsequently, sections were rinsed in Flex wash buffer (K8007; Agilent) and loaded onto the autostainer. Peroxidase blocking was performed (S2023; Agilent) and slides were rinsed with the Flex wash buffer again. Slides were incubated with the vimentin antibody (Cell Signaling #5741) diluted 1/100 in antibody diluent (S2023; Agilent) for 40 min at room temperature. The sections were then rinsed with Flex wash buffer before application of the Rabbit Envision secondary antibody (K4003; Agilent) for 30 min at room temperature. Sections were subsequently rinsed with Flex wash buffer before applying Liquid DAB (K3468; Agilent). The sections were finally washed in water, counterstained with Haem Z, dehydrated in graded ethanol, placed in xylene and coverslipped using DPX mountant.

All slides were imaged using a Zeiss AX10 light microscope at magnifications listed in the figure legends.

AB/PAS and Ki67 staining of pancreas tissue were quantified as markers of PanIN and cell proliferation, respectively. Histology slides of the pancreas tissues (of Alcian blue and Ki67) were scanned with the Aperio AT2 slide scanner (Leica Biosystems, UK) at 20X magnification and quantified using the HALO image analysis platform (Indica Labs, Albuquerque, NM, USA). Alcian blue/PAS staining was quantified using the area quantification v2.4.2 algorithm. The data are presented as percentage of Alcian blue‐positive tissue over total area of the pancreas section. Ki67 staining was quantified using the Cytonuclear v2.0.9 algorithm. The data are presented as percentage of Ki67‐positive cells over total number of cells within the pancreas section. The number of mice counted for each analysis is specified in the respective figure legends.

### 2.2 Human samples

Samples were collected as part of the ADEPTS study led by Prof Stephen Pereira (Research Ethics Committee reference: 06/Q0512/106, IRAS Number 234637, Sponsor reference number 06/0153, NIHR portfolio number 7343). Human serum samples were collected at the Royal Free Hospital, London, and at University College London Hospitals (UCLH) from patients presenting with benign symptomatic pancreaticobiliary diseases (used as the controls in the analysis) or early‐stage (stage IA, IB, IIA and IIB) PDAC. Samples were collected from November 2018 to October 2022. The benign symptomatic pancreaticobiliary disease patients did not include any with chronic pancreatitis but included those admitted to hospital due to symptoms or other medical issues attributable to the pancreaticobiliary system. Patient characteristics are listed (Table 1). CA19‐9 levels were measured for 77 of the patients using the Cobas system (Roche, Basel, Switzerland), as previously published [18]. The experiments were undertaken with the understanding and written consent of all patients, and the study methods conformed to the standards set by the Declaration of Helsinki and were approved by the local ethics committee (UCL/UCLH).

**Table 1:** Demographic characteristics of the human patient serum samples analysed in this study: benign symptomatic group (controls) and early‐stage PDAC cases. Patient characteristics: the mean age ± standard deviation (SD) of the group in years, the sex (Male [M] and Female [F]) and also, disease stage for pancreatic ductal adenocarcinoma (PDAC) patients. Symptomatic benign pancreaticobiliary disease cases did not include chronic pancreatitis patients, but included those with: gallstone disease, gastritis/reflux disease, sphincter of Oddi dysfunction, familial pancreatic cancer, other biliary duct disease, deranged liver function test, abdominal pain, irritable bowel syndrome, as well as some with medical problems associated with the digestive syndrome (weight loss, constipation, longstanding iron deficiency anaemia) and other diagnoses such as mild‐dysphagia, a retroperioneal non‐cancerous mass and a duodenal polyp.

Characteristic	Symptomatic benign pancreaticobiliary disease cases (controls) (*n* = 62)	Early‐stage PDAC patients (*n* = 31)
Age, mean ± SD (years)	71 ± 10.4	71 ± 10.1
Sex, *n* (%):		
Male	26 (41.9%)	15 (48.4%)
Female	36 (58.1%)	16 (51.6%)
Cancer stage, *n* (% of the PDAC cases) (Male (M)/Female (F)):		
IA	‐	8 cases (25.8%) 2 m/6 F
IB	‐	7 cases (22.6%) 1 m/6 F
IIA	‐	4 cases (12.9%) 3 m/1 F
IIB	‐	12 cases (38.7%) 9 m/3 F

### 2.3 Proteomics sample preparation

On a plate shaker at 1400 rpm, aliquots of mouse or human serum (diluted 1 : 10 in 0.1 m Tris [pH 7 or 8, respectively], 2% sodium deoxycholate detergent) were reduced using 5 mm dithiothreitol (DTT) first at 95 °C for 5 min and then for 30 min at 25 °C. Reduced proteins were then alkylated using 55 mm iodoacetamide (IAA) at 25 °C for 1 h in the dark.

For protein digestion, an Agilent AssayMAP Bravo equipped with 96LT head and a ‘Magnetic bead accessory’ plate was used with a custom script provided from Agilent. Alkylated proteins were mixed with conditioned magnetic hydroxyl beads (Resyn Bioscience, Gauteng, South Africa) and left to aggregate for 20 min. Beads were washed in 80% ethanol and supernatant was discarded. Protein bead aggregates were finally resuspended in 100 mm ammonium bicarbonate (AMBIC), and trypsin (Promega, Madison, WI, USA) was added with an enzyme : substrate ratio of 1 : 200. The plate was removed from the AssayMAP Bravo and samples were incubated at 37 °C on a shaker at 1500 rpm. Using the AssayMAP Bravo, equipped with a 96 AM head, samples were acidified to 1% trifluoroacetic acid (TFA), and finally desalted using AssayMAP 5 μL C18 cartridges (Agilent) with the ‘peptide cleanup 2.0’ application used with default settings.

### 2.4 Proteomics liquid chromatography‐mass spectrometry (LC–MS)

Proteomic analysis on all serum samples was carried out using a reverse‐phase C18 column on an EASY‐nLC II 1200, coupled to a Q‐Exactive HF mass spectrometer. Elution of peptides (at 300 nL·min over 75 min) was conducted with a binary gradient with buffer A (2% acetonitrile with 0.1% formic acid) and B (80% acetonitrile with 0.1% formic acid), with: 1 min at 3% B, 42 min at 23% B, 14 min at 38% B, 9.5 min at 95% B and the final 4.5 min at 3% B. Samples were loaded alongside buffer A into a 50‐cm fused silica emitter (New Objective, Woburn, MA, USA) packed in‐house with ReproSil‐Pur C18‐AQ, 1.9 μm resin (Dr Maisch GmbH, Ammerbuch, Germany). The packed emitter was held at 50 °C using a column oven (Sonation) integrated into the nano‐electrospray ion source (Thermo Scientific, Waltham, MA, USA), which was used to electrospray eluting peptides online into the mass spectrometer. An Active Background Ion Reduction Device (ABIRD) (ESI Source Solutions, Woburn, MA, USA) was used to reduce ambient contaminant signal level.

For MS ionisation condition settings, the spray voltage was set to 2.1 kV and ion transfer tube temperature to 250 °C. xcalibur software (version 4.2.47, Thermo Scientific) was used for data acquisition, which was carried out in positive ion mode using data‐independent acquisition (DIA). A full scan (FT‐MS) over a mass range of 340–1050 m/z was acquired at 60 000 resolution, at 200 m/z, with a target value of 3 000 000 ions for a maximum injection time of 54 ms. Higher energy collisional dissociation fragmentation spectra were recorded at 30 000 resolution, at 200 m/z. All precursors were fragmented using 28 consecutive windows with 25 Da width, allowing for a 0.5 m/z overlap, covering a mass range from 350 to 1023 m/z. All ions were fragmented using a normalised collision energy of 28, for a maximum injection time of 54 ms, or a target value of 30 000 ions.

### 2.5 MS data processing

Data were processed in spectronaut version 18.2 [Mouse] or 19.0 [Human] (Biognosys, Schlieren, Switzerland) using UniProt databases (Canonical and isoform databases (13‐09‐2022) for Mus musculus [25 198 entries] and Homo sapiens [42 438 entries]), with the MaxQuant contaminant database used when needed (www.uniprot.org) [19]. Minimum peptide length was set to seven amino acids and specificity for trypsin/P cleavage was required, allowing up to two missed cleavage sites. Methionine oxidation and N‐terminal acetylation were specified as variable modifications, whereas carbamidomethylation of cysteine was set as a fixed modification.

Minor peptide grouping was set to ‘Modified Sequence’, and Major and Minor Group Quantity were set to ‘Sum peptide quantity’ and ‘Median precursor quantity’, respectively. Single hit proteins were excluded from the dataset and all other spectronaut parameters were left to their default options. Data from spectronaut for proteins and peptides were then analysed in perseus [version 1.6.14.0 and 1.6.15.0] and visualised in r studio [version 4.3.2] [20, 21]. If samples exceeded one plate, batch correction was applied in perseus using the combat batch effect reduction method [22].

In perseus, the intensity of the proteins was normalised by dividing by the median of all intensities within a sample and then proteins were filtered to only keep those present that had at least a certain percentage (70%) of valid values in each group in the screen. For the PanIN GEMM serum proteomics screen, this was eight groups: four genotypes across two ATG colonies (one in which ATG7 is deleted and the other where ATG5 is deleted) (Table S1). This meant only proteins present in at least 70% of every group of the eight separate groups were included in the analysis of that screen. For the KPC serum proteomics screen, the proteins needed to be in at least 70% of the samples of each group (wild‐type or KPC) to be included in the analysis. For the human serum proteomics screens, the proteins needed to be in at least 70% of the samples of each group (controls or early‐stage PDAC patients) to be included in the analysis. Any missing values were imputed separately for each column (each serum sample) using a normal distribution imputation with parameters set to 0.3 standard deviations for ‘width’ and 1.8 standard deviations for ‘down‐shift’. Data were log2 transformed and subsequently analysed as detailed in the ‘Data analysis and statistics’ section below.

### 2.6 Data analysis and statistics

#### 2.6.1 perseus (version 1.6.15.0)

Data from the proteomics screens were processed in perseus. However, prior to analysis, data from all the samples were plotted in histograms (one analyte per histogram) and visually checked for normal distribution.

For the initial serum proteomics screen on the PanIN GEMMs, the eight groups of mice (four genotypes across the two ATG colonies) were compared using a one‐way analysis of variance (ANOVA) with a permutation‐based false discovery rate (FDR) at 0.05, with 250 randomisations. Proteins were filtered to keep those with significance and then a Post hoc Tukey's honest significant difference (HSD) test for one‐way ANOVA was conducted (FDR at 0.05) to assess which groups of mice the hits were significant between.

For determining which significant hits were specific to the PanIN GEMMs, hits from the above analysis were filtered to keep only those significant between wild‐type and PanIN GEMMs but discarding any that showed significance between wild‐type and Cre‐only mice, between wild‐type and impaired pancreatic autophagy mice or between Cre‐only and impaired pancreatic autophagy mice. This is because any hits caused by Cre alone are off‐target and any caused by impaired pancreatic autophagy were not of interest for the project. The filtering was conducted for each ATG colony separately, giving a PanIN‐specific hit list for each. The two hit lists were then compared, and the results were plotted using biovenn (www.biovenn.nl).

For the screens on the KPC mice and on the patient samples, Student's *t*‐test with a permutation‐based FDR at 0.05, with 250 randomisations was used. This compared KPC mice to wild‐type mice and early‐stage PDAC patients to benign pancreaticobiliary disease cases (controls), respectively. Proteins from each were then filtered for significance. Overlap was assessed and subsequently visualised using biovenn.

#### 2.6.2 r studio (version 4.3.2 to 4.5.1)

r studio was used to generate plots of the serum proteomics screen datasets [21]. For each screen, principal component analysis (PCA) plots [using the ggplot function from the ggplot2 package, version 3.5.1] were used to help visualise the large multidimensional data. Heatmaps of the significant hits in each screen were generated [using the heatmap function from the complexheatmap package, version 2.16.0], after z‐scores had been calculated in perseus. Heatmap rows were separated into those significantly increasing or decreasing and subsequently clustered with these groups, using Spearman's rank correlation.

#### 2.6.3 string database (version 12.0) enrichment analysis

Enrichment analysis of the gene names of the significant protein hits from the serum proteomics screens was conducted using the Search Tool for the Retrieval of Interacting Genes/Proteins (string) Database (www.string‐db.org) [23]. The analysis setting for the minimum required interaction score for it to be included in the results was set to 0.4 [string terms this as ‘medium confidence’]. The analysis parameters deem the confidence as the estimated probability that a predicted interaction occurs between two proteins in the network in the database [using the Kyoto Encyclopaedia of Genes and Genomes (KEGG) pathway database] [23].

There are three readouts as part of the enrichment results: count in network, strength and FDR. The count in network is the number of protein hits in the hitlist that appear in that group (e.g. that KEGG pathway) out of the total number of proteins that belong to that group (that KEGG pathway). The strength is a number representing the strength of the enrichment; it is calculated by using the formula: Log10 (observed/expected). This is the log [to the base 10] of the number of hits observed in that group (KEGG pathway) divided by the number of hits that you would expect to be found in that pathway in a random list of proteins that has the same number of proteins as your input list of protein hits. The FDR is the p‐value for each of the results, which were corrected for multiple hypothesis testing with the Benjamini–Hochberg method.

#### 2.6.4 University of Alabama at Birmingham cancer (UALCAN) data analysis portal

The UALCAN data analysis portal was used to assess the potential relevance of the significant hits seen in the serum proteomics screens, in terms of their levels in human PDAC tumours [24, 25]. Analysis in the portal compared normal tissue to PDAC tissue from stages 1–4, using the Clinical Proteomic Tumour Analysis Consortium (CPTAC) Pancreatic Adenocarcinoma dataset, plotting the z‐value [standard deviations from the median of all samples] for each sample (74 normal samples, and then 4 stage 1, 65 stage 2, 35 stage 3 and 7 stage 4 pancreatic adenocarcinoma samples). The website plots the data as box‐and‐whisker plots, where the box represents the interquartile range [spanning from the lower quartile (Q1) to the upper quartile] with a black line representing the median. The whiskers extend to the minimum and maximum z‐values for that group of samples. Statistical analysis is built into the portal and compares the normal tissue to each stage (1–4) of pancreatic adenocarcinoma.

#### 2.6.5 cbioportal (version v6.0.25)

cbioportal was used to assess whether there was a correlation between hits identified in the proteomics screens, in terms of the levels of the proteins in individual PDAC patients. Data from the ‘Pancreatic Ductal Adenocarcinoma (CPTAC, Cell 2021)’ dataset was used [26]. Proteins were plotted in pairs (using their ‘protein abundance ratio z‐scores’) and a regression line was plotted in cbioportal, alongside the statistics which used Pearson's correlation to generate a p‐value for the correlation between the two proteins in the plot. Data points were also coloured based on sex (males as blue and females as pink).

### 2.7 Analysis of a published human PDAC proteomics dataset

Published tissue proteomics data was assessed for presence of hits identified in this study. The data includes human patient samples of normal, PanIN and PDAC tissue [27]. Data from Table S1 in the paper was used, filtered for specific proteins of interest and subsequently a heatmap was generated [using the heatmap function from the complexheatmap package, version 2.16.0 as explained in the ‘r studio’ section above].

### 2.8 Receiver operating characteristic (ROC) curves

ROC curves of data from the early‐stage PDAC patient serum proteomics screen combined with CA19‐9 values (where available) were generated using metaboanalyst (version 6.0) (www.metaboanalyst.ca), with the ‘Biomarker analysis’ module on the website. The ROC analysis option ‘ROC curve‐based model evaluation’ was used and all parameters on the website were left to their default settings, using the ‘Linear SVM’ algorithm option. Proteins were selected individually or combined with each other to generate ROC curves of the data and assess the sensitivity or specificity in terms of distinguishing the early‐stage PDAC patients from the benign pancreaticobiliary disease cases (controls).

### 2.9 DepMap analysis

Data from DepMap from the drug screen dataset PRISM Repurposing Public 24Q2 (available at https://depmap.org/portal) [28, 29] were analysed to assess the correlation between the expression of different hits identified in our screen with the drug sensitivity to the KRAS inhibitor sotorasib in samples categorised as ‘pancreatic adenocarcinoma’ for primary disease (data version batch corrected Expression Public 24Q4). Expression of KRAS was also included in the analysis to identify any potential correlation of this with the expression of the different hits and the drug sensitivity.

### 2.10 Statistics

Results were deemed significant where *P* < 0.05 or FDR < 0.05 (depending on the analysis used). In figures, a * denotes a significant result, whereas ns denotes that the observation was not significant. For IHC quantification, statistical analysis was performed using the unpaired Student's *t*‐test (graphpad prism 10.2.2). ns, not significant; *P < 0.05; **P < 0.01; ***P < 0.001.

## 3 Results

### 3.1 Mice with abundant PanINs show a variety of significant changes in their serum proteome

**Fig. 1:**
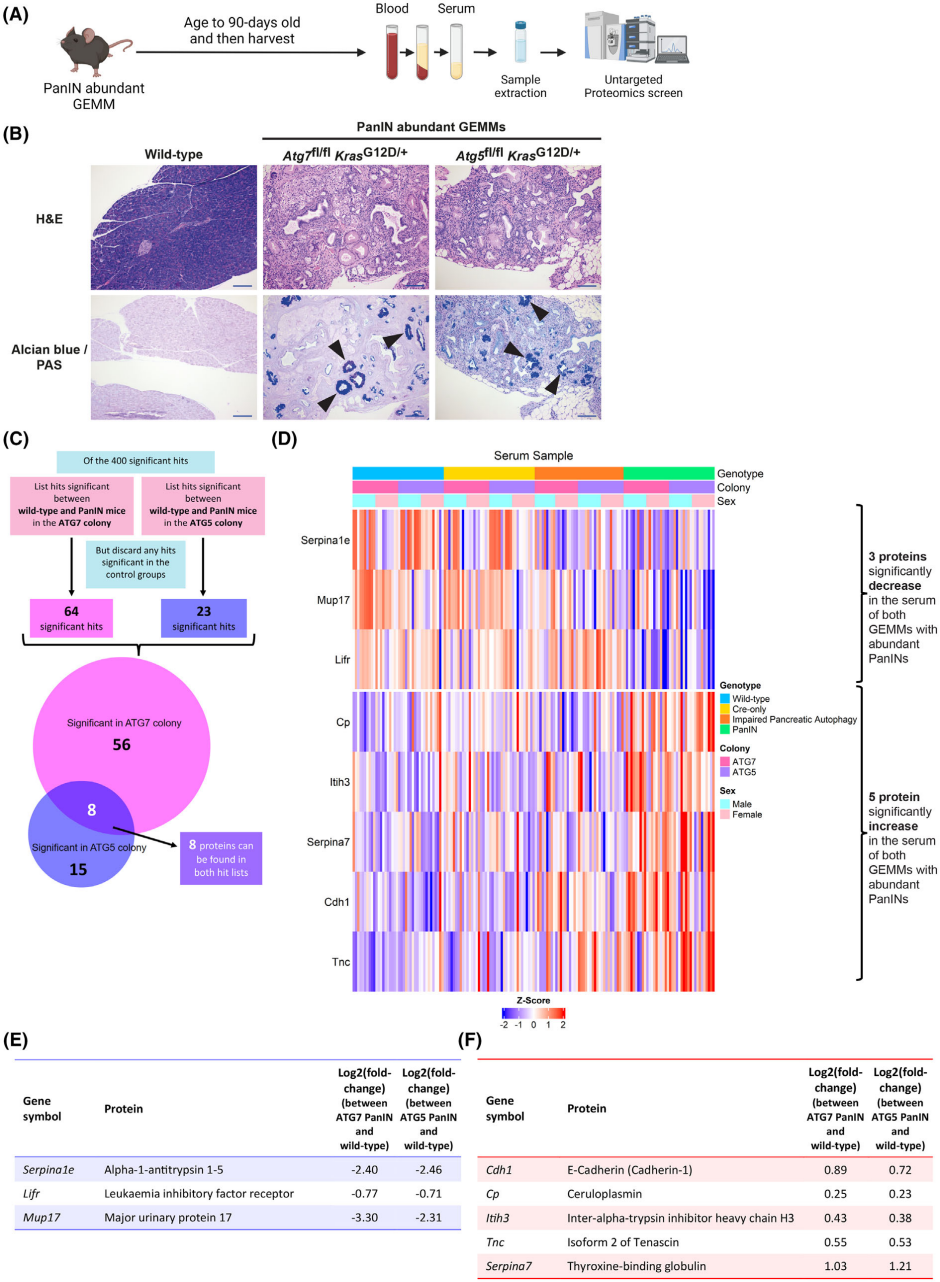
Serum proteomics changes in the PanIN‐abundant GEMMs. (A) Schematic of sample collection for the pancreatic intraepithelial neoplasm (PanIN)‐abundant genetically engineered mouse models (GEMMs) (Created in BioRender. Mearns, H. (2025) https://BioRender.com/i94f367). (B) Representative pancreata histology images (*n* = 20 for each genotype) of haematoxylin and eosin (H&E) and Alcian blue/Periodic Acid‐Schiff (PAS) of wild‐type mice and both PanIN‐abundant GEMMs (*Pdx1‐Cre Atg7* ^fl/fl^ *Kras* ^G12D/+^ and *Pdx1‐Cre Atg5* ^fl/fl^ *Kras* ^G12D/+^) at 90‐days (+/− 10 days) old. Arrows indicate PanINs. Images at 10X magnification with 500 μm scale bars shown. The wild‐type H&E and Alcian blue/PAS images are also used as reference in Figs S2B and S1A, as they are the same reference samples. (C) Schematic of filtering of the significant hits in the two PanIN‐abundant GEMMs: 64 in the ATG7 PanIN mice and 23 hits in the ATG5 PanIN mice, with 8 hits overlapping between the two. (D) Heatmap of the 8 significant serum proteins in the PanIN‐abundant GEMMs: 3 proteins were significantly decreased, and 5 proteins were significantly increased (gene symbols are shown on the left‐hand side of the heatmap) (z‐score blue (−2) to red (2), across 159 serum samples: 10 males and 10 females of each genotype, in each ATG colony (except 9 males for the ATG7 impaired pancreatic autophagy group)). (E) Table of the 3 serum proteomics hits that decrease in the PanIN GEMMs, their gene symbols, and their log2 (fold‐changes). (F) Table of the 5 serum proteomics hits that increase in the PanIN GEMMs, their gene symbols, and also their log2 (fold‐changes) compared to their wild‐type counterparts.

To analyse the impact of abundant PanINs on the serum proteome, mice were generated with a heterozygous *Kras* ^G12D^ mutation, alongside the loss of either *Atg7* or *Atg5*. This was achieved through the use of floxed alleles (or a floxed stop codon upstream of the *Kras* ^G12D^) and a Cre recombinase under the control of the *Pdx1* promoter, which is primarily expressed in the pancreas and duodenum, leading to greatly exacerbated PanIN formation (as quantified as PanINs numbers per mm^2^ of pancreatic tissue) in the pancreas of these animals [16, 30]. The serum proteome of these abundant PanIN GEMMs had not been previously characterised, hence it was of particular interest to assess these mice for proteomic changes in an unbiased manner (Fig. 1A).

The presence of abundant PanINs in the pancreatic tissue of the PanIN‐abundant mice, for both ATG colonies can be seen following H&E staining as well as staining for mucin produced by PanINs, using Alcian blue/PAS (Fig. S1A,B) as was previously published [16]. These analyses revealed that macroscopically, the impaired pancreatic autophagy mice showed little difference from their wild‐type and Cre‐only counterparts (Fig. S1A).

Proteomic comparison of serum samples from the various genotypes of mice at 90 days [wild‐type mice, Cre‐only mice (*Pdx1‐Cre*), impaired pancreatic autophagy mice (*Atg7* ^−/−^ or *Atg5* ^−/−^, with *Pdx1‐Cre*) and PanIN‐abundant mice (*Atg7* ^−/−^ or *Atg5* ^−/−^, with *Kras* ^G12D/+^ and *Pdx1‐Cre*)] across the ATG7 and ATG5 colonies revealed that mice with abundant PanINs had significant changes compared to wild‐type controls. The hit lists of proteins in the serum with significant changes were generated separately for each ATG colony of PanIN mice (ATG7 and ATG5) and were identified following removal of any significant hits seen in Cre‐only mice or the impaired pancreatic autophagy mice that do not form PanINs (*Atg7* ^−/−^, *Pdx1‐Cre* or *Atg5* ^−/−^, *Pdx1‐Cre*, respectively) (Tables S2 and S3). This filtering was conducted to help ensure that the results taken forward were due to the presence of the PanINs in these mice and not the underlying impaired autophagy in their pancreata, as was previously published [16].

Subsequent analysis revealed that of the significant hits seen in the two colonies of PanIN‐abundant mice (ATG7 and ATG5), eight proteins showed significant changes in both models and these proteins also changed in the same direction in both colonies (Fig. 1C). The heatmap shows the z‐scores across the different serum samples of the various genotypes of mice, for the eight proteins of interest (Fig. 1D). Of these eight proteins, three showed a significant decrease in the mice with abundant PanINs, whereas five proteins showed a significant increase in this group compared to the wild‐type controls (Fig. 1E,F). The three decreasing proteins were: alpha‐1‐antitrypsin 1–5, leukaemia inhibitory factor receptor and major urinary protein 17. The five increasing proteins were: E‐cadherin, ceruloplasmin, inter‐alpha‐trypsin inhibitor heavy chain H3 (ITIH3), tenascin and thyroxine‐binding globulin. E‐cadherin and tenascin are glycoproteins involved in cell–cell adhesion and the extracellular matrix (ECM), respectively [31, 32]. Ceruloplasmin, initially identified to be synthesised by hepatic cells, has been shown to be secreted from Capan‐1 PDAC cells [33, 34]. ITIH3 is a protein that is part of the pre‐alpha‐trypsin inhibitor complex and helps to stabilise the ECM by interacting with hyaluronan [35]. Lastly, the role of thyroxine‐binding globulin is to bind thyroid hormones in the serum [36].

Dimensionality reduction of the data by principal component analysis (PCA) (Fig. S1C) revealed that there was little difference in the overall serum proteome between the various genotypes of mice used in the screen, despite the significant differences seen in specific proteins in the serum. This suggests that the abundant PanINs are not altering the overall serum proteome but are instead influencing (directly or indirectly) the levels of specific proteins.

### 3.2 KPC mice show significant changes in their serum proteome

**Fig. 2:**
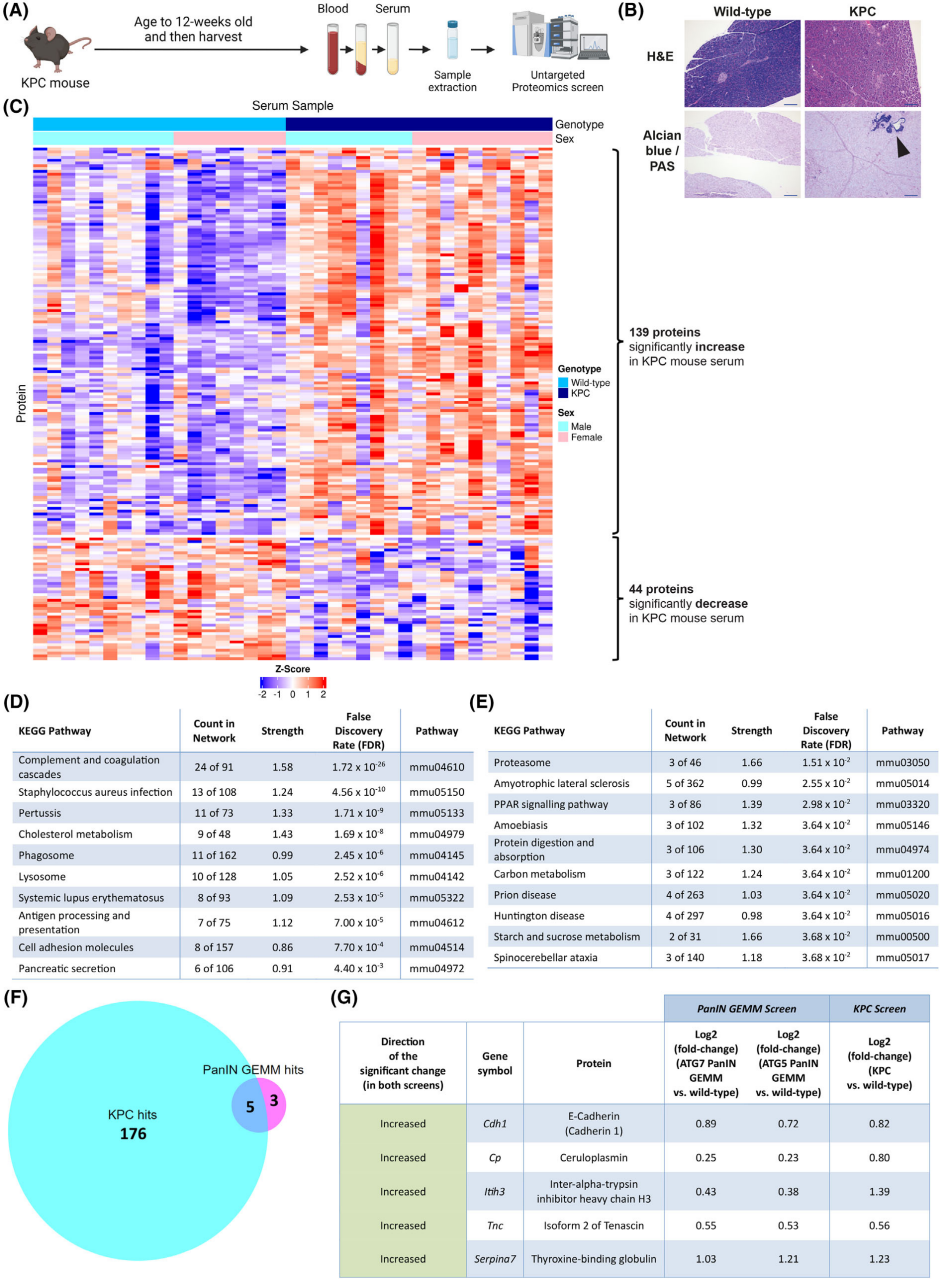
Serum proteomics changes in the pre‐pancreatic ductal adenocarcinoma (PDAC) KPC mice. (A) Schematic of sample collection for the KPC (*Kras* ^G12D/+^ *Trp53* ^R172H/+^ *Pdx1‐Cre*) mice (Created in BioRender. Mearns, H. (2025) https://BioRender.com/qy6c46p). (B) Representative pancreata histology images (wild‐type, *n* = 18, KPC, *n* = 19) of haematoxylin and eosin (H&E) and Alcian blue/Periodic Acid‐Schiff (PAS) of wild‐type mice and KPC mice (*Kras* ^G12D/+^ *Trp53* ^R172H/+^ *Pdx1‐Cre*) at 12‐weeks (+/− 10 days) old. Arrow indicates pancreatic intraepithelial neoplasms (PanINs). Images at 10X magnification with 500 μm scale bars shown. The wild‐type H&E and Alcian blue/PAS images are also used as reference in Fig. S1A,B as they are the same reference samples. (C) Heatmap of the 183 serum proteomics hits in the KPC mice: 139 were significantly increased (upper group of rows in the heatmap) and 44 were significantly decreased (lower group of rows in the heatmap) (z‐score blue (−2) to red (2), across 37 serum samples: 18 wild‐type (10 males and 8 females) and 19 KPC (9 males and 10 females) samples). (D) Top 10 significantly enriched Kyoto Encyclopaedia of Genes and Genomes (KEGG) pathways in the increased hits in the KPC mice. (E) Top 10 significantly enriched KEGG pathways in the decreased hits in the KPC mice. (F) Venn diagram of the overlap of 5 proteins between the significant serum proteomics hits in the PanIN genetically engineered mouse model (GEMM) and the KPC screens: 8 proteins and 181 proteins (from the 183 hits seen), respectively. (G) Table of the 5 overlapping increasing serum proteomics hits between the PanIN GEMMs and KPC, with their respective log2 (fold‐changes) listed (compared to their wild‐type counterparts).

To assess the impact of lower PanIN abundance in KPC mice on the serum proteome, and to investigate whether similar significant changes could be seen as observed in the PanIN‐abundant mice, KPC (*Kras* ^G12D/+^ *Trp53* ^R12H/+^ *Pdx1‐Cre*) mice were aged to 12 weeks, harvested, and blood taken for serum analysis (Fig. 2A). Histological analysis of the pancreata revealed that 12‐week‐old KPC mice harbour a small number of PanINs, but rarely PDAC (Fig. 2B). Moreover, PCA of the KPC serum proteomics revealed that the KPC mice cluster away from the wild‐type mice, in terms of their overall serum proteome (Fig. S2A).

Proteomic analysis comparing samples from KPC and wild‐type mice revealed that 183 hits (accounting for 181 different proteins) were significantly different between the two genotypes. The heatmap in Fig. 2C shows the changes in z‐scores of the 183 hits across the serum samples. Of the significant hits, 139 showed significant increases in the KPC mice, with 44 showing significant decreases in this group (Table S4).

To investigate the potential biological relevance of the significant changes seen in the KPC mouse serum proteome, enrichment analysis was performed using the string Network platform for each list, with significantly decreased and increased hits analysed separately. Of the 139 protein hits showing a significant increase, and after two duplicate protein names were removed, 127 of the hits were available on the string database. Network analysis of these hits revealed that there was significant enrichment in various KEGG pathways, including: ‘complement and coagulation cascades’, ‘cholesterol metabolism’ and ‘pancreatic secretion’ (Fig. 2D).

Enrichment analysis of the proteins showing significant decreases (31 of the 44 proteins were available on the string database) revealed significant enrichment in 12 KEGG pathways. The top 10 enriched pathways are plotted in Fig. 2E and included: ‘carbon metabolism’ and ‘peroxisome proliferator‐activated receptors (PPAR) signalling’.

To assess for similarities between the PanIN‐abundant GEMMs and the 12‐week‐old KPC mice, the hit lists from the two serum proteomic screens were compared. This revealed that the five proteins seen to be significantly changed in the PanIN‐abundant GEMMs were also present in the KPC mouse significant hits list (Fig. 2F). These hits were: E‐cadherin, ceruloplasmin, ITIH3, tenascin and thyroxine‐binding globulin. Analysis revealed that all five hits were significantly increased in both the PanIN‐abundant GEMMs and the KPC mice, with the log2 fold‐changes shown (Fig. 2G). This suggests that these five hits are associated with PanIN formation in both mouse models. To further assess cell proliferation in the pancreatic tissue across the genotypes of mice, additional analysis of Ki67‐positive nuclei was performed. This revealed that both males and females showed increased staining in PanIN‐abundant GEMMs and KPC mice when compared to the wild‐type counterparts (Fig. S2B).

### 3.3 Early‐stage PDAC patients show significant changes in their serum proteome compared to symptomatic patients with benign disease

**Fig. 3:**
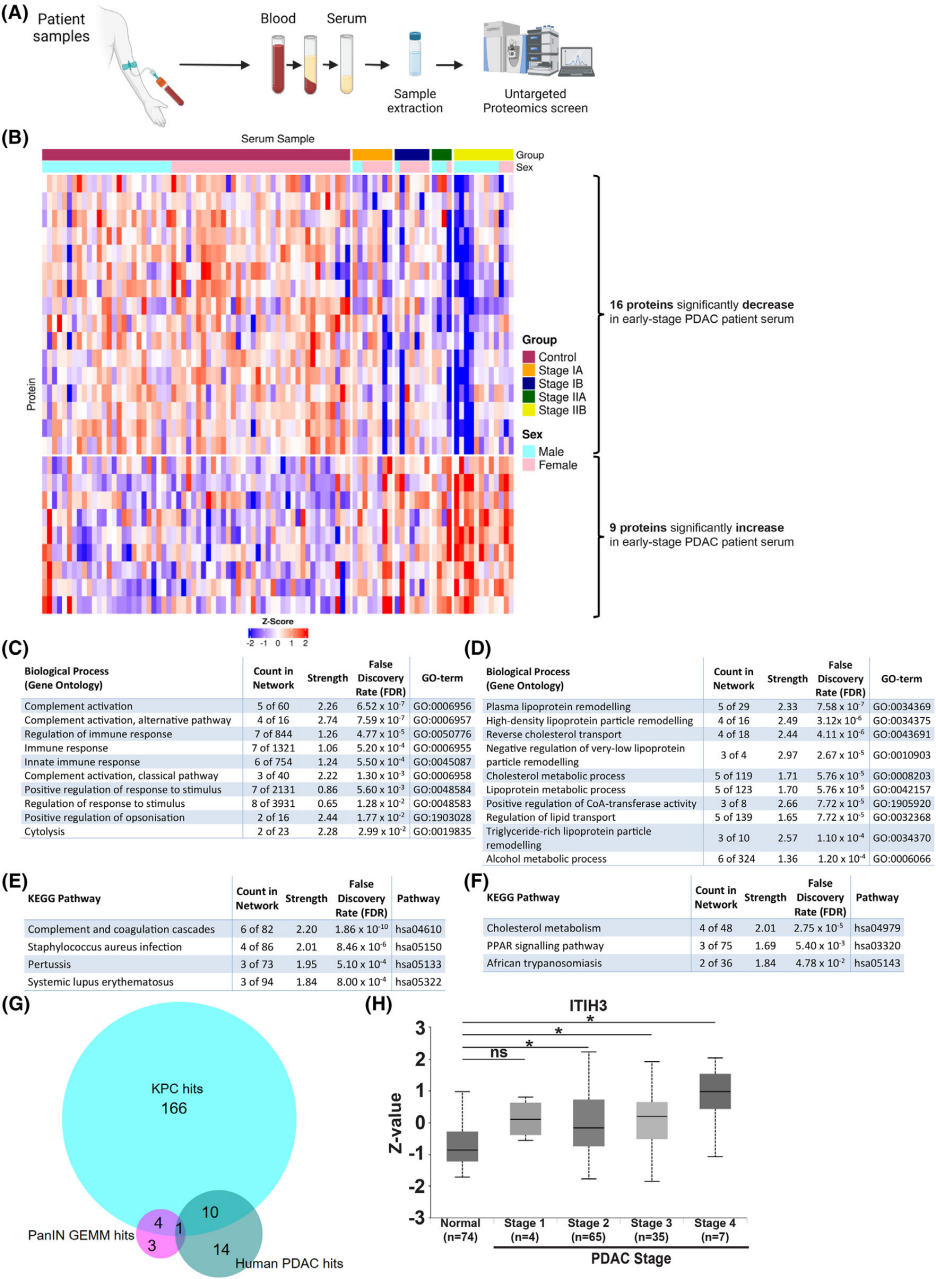
Serum proteomics changes in early‐stage PDAC patients (compared to benign symptomatic pancreaticobiliary disease case controls). (A) Schematic of sample collection for the early‐stage pancreatic ductal adenocarcinoma (PDAC) patients and controls (Created in BioRender. Mearns, H. (2025) https://BioRender.com/45nspea). (B) Heatmap of the 25 serum proteomics hits in the early‐stage PDAC patients: 9 proteins were significantly increased (lower group of rows in the heatmap) and 16 were significantly decreased (upper group of rows in the heatmap) (z‐score blue (−2) to red (2), across 93 serum samples: 62 symptomatic controls and 31 early‐stage PDAC patients). (C) Top 10 significantly enriched biological processes in the increased hits in the early‐stage PDAC patients. (D) Top 10 significantly enriched biological processes in the decreased hits in the early‐stage PDAC patients. (E) 4 significantly enriched Kyoto Encyclopaedia of Genes and Genomes (KEGG) pathways in the increased hits in the early‐stage PDAC patients. (F) 3 significantly enriched KEGG pathways in the decreased hits in the early‐stage PDAC patients. (G) Venn diagram of the overlap between the mouse and human serum proteomics screens: eight proteins in the pancreatic intraepithelial neoplasm (PanIN) genetically engineered mouse models (GEMMs), 181 proteins (from 183 hits seen) in the KPC (*Kras* ^G12D/+^ *Trp53* ^R172H/+^ *Pdx1‐Cre*) mice and 25 proteins in the human PDAC patients. (H) Box plot of protein expression of inter‐alpha‐trypsin inhibitor heavy chain H3 (ITIH3) across normal tissue and pancreatic adenocarcinoma tumour stages 1–4 (using the Clinical proteomic tumour analysis consortium (CPTAC) Pancreatic Adenocarcinoma dataset on the University of Alabama at Birmingham Cancer (UALCAN) data analysis portal). Box‐and‐whisker plot is shown, where the box represents the interquartile range [spanning from the lower quartile (Q1) to the upper quartile] with a black line representing the median, with Welch's *t*‐test used for the statistics. * (*P* < 0.05), ns (not significant).

To assess whether the findings seen in the PanIN‐abundant GEMMs and the KPC mice could be translated to humans, a proteomics screen comparing 31 serum samples taken from early‐stage (stage IA, IB, IIA and IIB) PDAC patients with 62 serum samples taken from symptomatic benign pancreaticobiliary disease cases (PDAC‐free) was conducted (Table 1 and Fig. 3A). A PCA plot generated using the patient serum proteomics data revealed that there was a degree of separation between the early‐stage PDAC patients and the symptomatic controls (Fig. S3A) in their overall serum proteomes.

Subsequent data analysis revealed that there were 25 proteins with significant differences between the serum of early‐stage PDAC patients and that of symptomatic controls. Of the 25 hits, 9 proteins showed significant increases, with 16 showing significant decreases in the PDAC patients when compared to the controls; z‐scores for the 25 proteins across the serum samples are shown in the heatmap included in Fig. 3B. A hit list for the significantly increased and decreased proteins in the early‐stage PDAC patients was generated (Table S5).

Enrichment analysis of the 9 hits showing significant increases revealed that there was a significant enrichment in various biological processes, including activation of both the complement cascade and the immune response (Fig. 3C,E). In contrast, enrichment analysis of the 16 significantly decreased proteins showed significant enrichment in different biological processes including ‘cholesterol metabolism’ and those linked to lipoprotein metabolism (Fig. 3D,F).

Comparison analysis of the three serum proteomics screens (PanIN‐abundant GEMMs, KPC mice and early‐stage PDAC patients) revealed that there was one hit that significantly increased across all three (Fig. 3G). This significant hit was ITIH3, which, as mentioned earlier, has a role in the ECM [35]. Analysis of a publicly available tissue proteomics dataset of pancreatic adenocarcinoma patients, from the clinical proteomic tumour analysis consortium (CPTAC) on the University of Alabama at Birmingham Cancer (UALCAN) data analysis portal, showed that ITIH3 is significantly increased in PDAC when compared to normal tissue and that this increase is associated with PDAC stage (Fig. 3H).

### 3.4 Overlap between the serum proteome hits in the GEMMs and early‐stage PDAC patients

**Fig. 4:**
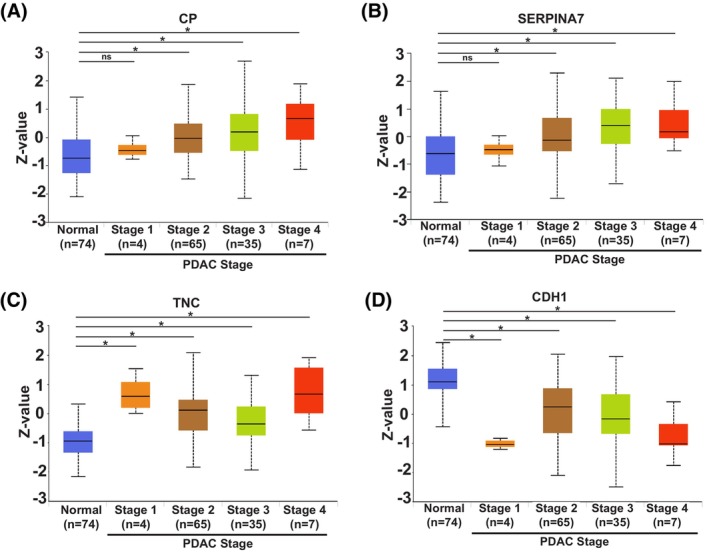
Human pancreatic adenocarcinoma tissue protein expression of the serum proteomics hits overlapping between pancreatic intraepithelial (PanIN) genetically engineered mouse models (GEMMs) and KPC (*Kras* ^G12D/+^ *Trp53* ^R172H/+^ *Pdx1‐Cre*) mice associates with tumour stage, using the CPTAC Pancreatic Adenocarcinoma dataset on the UALCAN analysis portal. Box plots of protein expression across normal tissue and pancreatic adenocarcinoma tumour stages 1–4 (using the Clinical proteomic tumour analysis consortium (CPTAC) Pancreatic Adenocarcinoma dataset on the University of Alabama at Birmingham Cancer (UALCAN) data analysis portal), for: (A) Ceruloplasmin (CP), (B) Thyroxine‐binding globulin (SERPINA7), (C) Tenascin (TNC) and (D, E) Cadherin (CDH1). Box‐and‐whisker plots are shown, where the box represents the interquartile range [spanning from the lower quartile (Q1) to the upper quartile] with a black line representing the median, with Welch's *t*‐test used for the statistics. * (*P* < 0.05), ns (not significant).

Following the serum proteomic screens, analysis revealed that of the significant proteins identified in each mouse model, five proteins overlapped between the abundant PanIN GEMMs and KPC mice. These were: ceruloplasmin, E‐cadherin, ITIH3, tenascin and thyroxine‐binding globulin. Subsequent analysis of a publicly available tissue proteomic dataset of pancreatic adenocarcinoma patients [Clinical Proteomic Tumour Analysis Consortium (CPTAC)] on the University of Alabama at Birmingham Cancer (UALCAN) data analysis portal revealed that four of these five proteins were significantly increased in the tumour tissue of patients (across the stages indicated) when compared to normal tissue from controls (Fig. 4A–C), whereas E‐cadherin was significantly decreased in the pancreatic adenocarcinoma tissues when compared to the control samples (Fig. 4D).

Analysis of the hit lists of significant proteins across the three screens revealed that although only one hit was found in all screens (ITIH3), there was also partial overlap between the early‐stage PDAC patients and the KPC mice. There were 11 hits that showed overlap between these two screens (Fig. 3G) and, of these 11 hits, five changed in the same direction, showing significant increases in both groups when compared to their respective controls. These hits were: complement C5 (C5), complement factor B (CFB), complement factor H (CFH), ITIH3 and monocyte differentiation antigen CD14 (CD14).

**Fig. 5:**
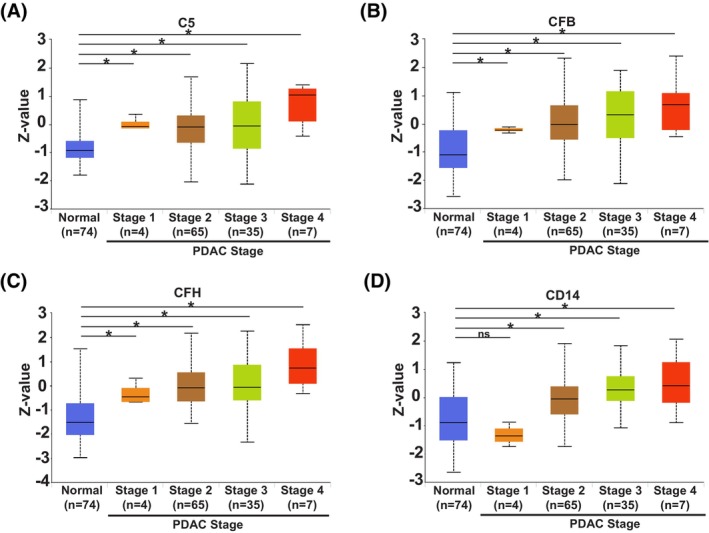
Human pancreatic adenocarcinoma tissue protein expression of the serum proteomics hits overlapping between KPC (*Kras* ^G12D/+^ *Trp53* ^R172H/+^ *Pdx1‐Cre*) mice and early‐stage pancreatic ductal adenocarcinoma (PDAC) patients associates with tumour stage, using the CPTAC Pancreatic Adenocarcinoma dataset on the UALCAN analysis portal. Box plots of protein expression across normal tissue and pancreatic adenocarcinoma tumour stages 1–4 (using the Clinical proteomic tumour analysis consortium (CPTAC) Pancreatic Adenocarcinoma dataset on the University of Alabama at Birmingham Cancer (UALCAN) data analysis portal), for: (A) Complement C5 (C5), (B) Complement factor B (CFB), (C) Complement factor H (CFH) and (D) Monocyte differentiation antigen CD14 (CD14). Box‐and‐whisker plots are shown, where the box represents the interquartile range [spanning from the lower quartile (Q1) to the upper quartile] with a black line representing the median, with Welch's *t*‐test used for statistical test. * (*P* < 0.05), ns (not significant).

Additional analysis, again using the pancreatic adenocarcinoma patient tissue proteomic dataset (CPTAC) on the UALCAN data analysis portal, revealed that these proteins were significantly increased in the tumour tissue of patients (across the stages indicated) when compared to normal tissue from controls (Figs 3H and 5A–D), suggesting their involvement in PDAC development and progression.

**Fig. 6:**
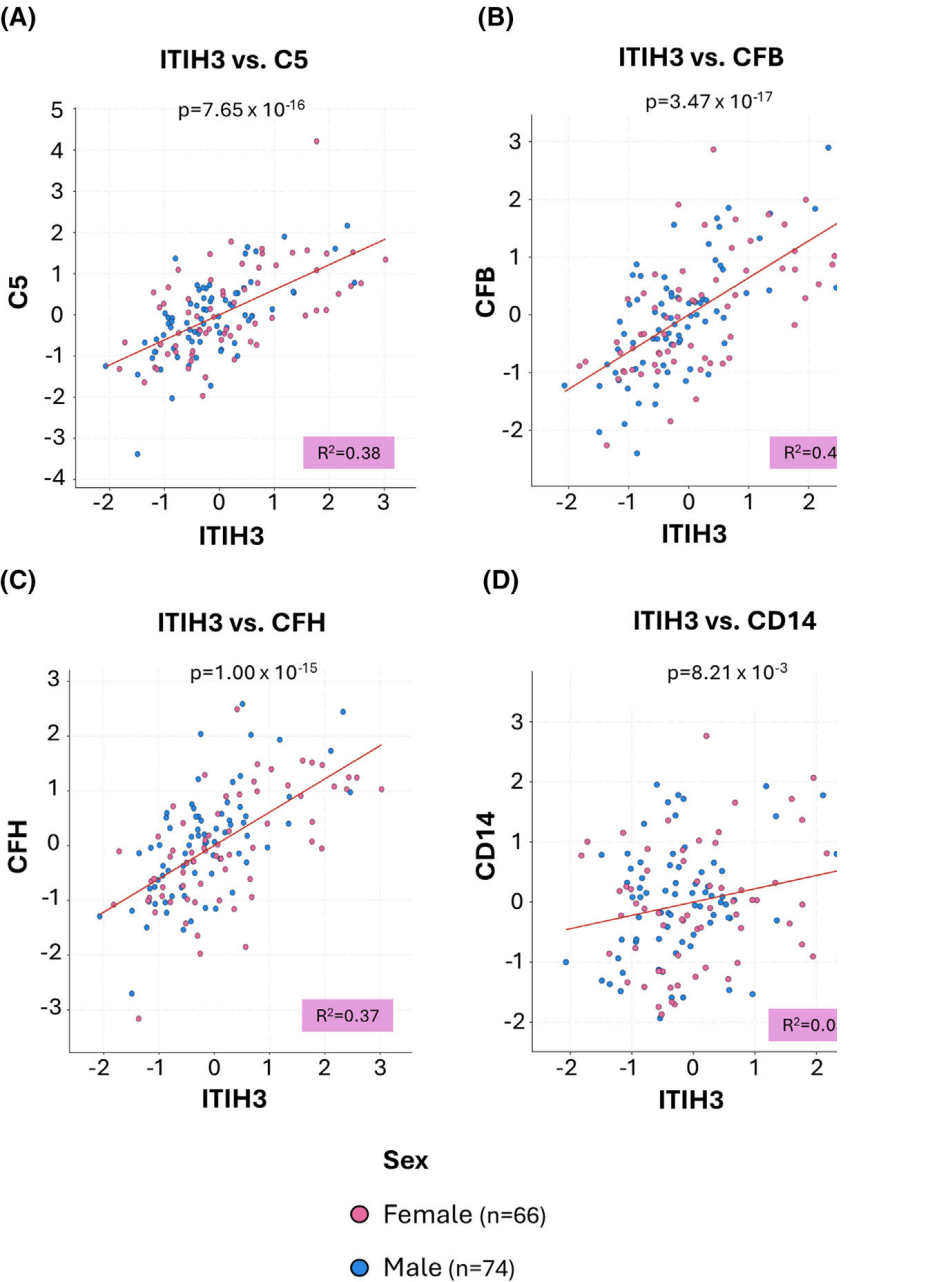
Positive correlation of tissue proteomics data in patients, of the serum hits overlapping between KPC (*Kras* ^G12D/+^ *Trp53* ^R172H/+^ *Pdx1‐Cre*) mice and early‐stage pancreatic ductal adenocarcinoma (PDAC) patients, using the Pancreatic Ductal Adenocarcinoma (Clinical proteomic tumour analysis consortium (CPTAC), Cell 2021) dataset on cbioportal. Correlations between inter‐alpha‐trypsin inhibitor heavy chain H3 (ITIH3) and the other protein hits, across patients: (A) ITIH3 vs. Complement C5 (C5), (B) ITIH3 vs. Complement factor B (CFB), (C) ITIH3 vs. Complement factor H (CFH), and (D) ITIH3 vs. Monocyte differentiation antigen CD14 (CD14). Each data point is a patient: female (*n* = 66) and male (*n* = 74). Data points are coloured based on sex: female (pink) and male (blue). Axes show the ‘Protein abundance ratio z‐scores’ for the protein level profile of each hit. The regression line (in red), R squared value and Pearson's correlation coefficient *P*‐value are shown for each comparison, with the Pearson's correlation coefficient statistical test used.

Further correlative analysis in cbioportal with the CPTAC Pancreatic Adenocarcinoma dataset revealed that the five hits [ITIH3, C5, CFB, CFH and CD14] are positively correlated with each other, and these correlations are significant for all pairwise comparisons of the hits, with pairwise comparisons against ITIH3 shown for simplicity (Fig. 6A–D). Further analysis of a published human tissue proteomics dataset in PDAC patients confirmed that four out of five of the hits [CFH, C5, ITIH3 and CD14] increase from normal to PanIN tissue and PDAC (Fig. S3B). The fifth protein [CFB] was not seen in their screen.

To further assess the diagnostic potential of the hits identified in this screening, we first analysed the serum levels of CA19‐9, a widely used diagnostic biomarker in human PDAC (Fig. S3C). These results showed that the early‐stage PDAC cases have significantly higher serum levels of CA19‐9 compared to the benign pancreaticobiliary disease cases.

To explore the potential origin of the hits identified in this screen, we successfully carried out IHC for four out of the five hits [ITIH3, CD14, CFB and CFH] (Fig. S4). Results showed that CFB expression was increased in the PanIN regions of the pancreata (as shown by AB/PAS staining in serial sections) of pre‐PDAC KPC mice compared to the acinar regions. Similarly, ITIH3 and CFH also showed to be more highly expressed in the pancreas of these mice compared to that of wild‐type mice, both in acinar and PanIN regions. CD14 staining showed little difference between the wild‐type and pre‐PDAC KPC mice.

**Fig. 7:**
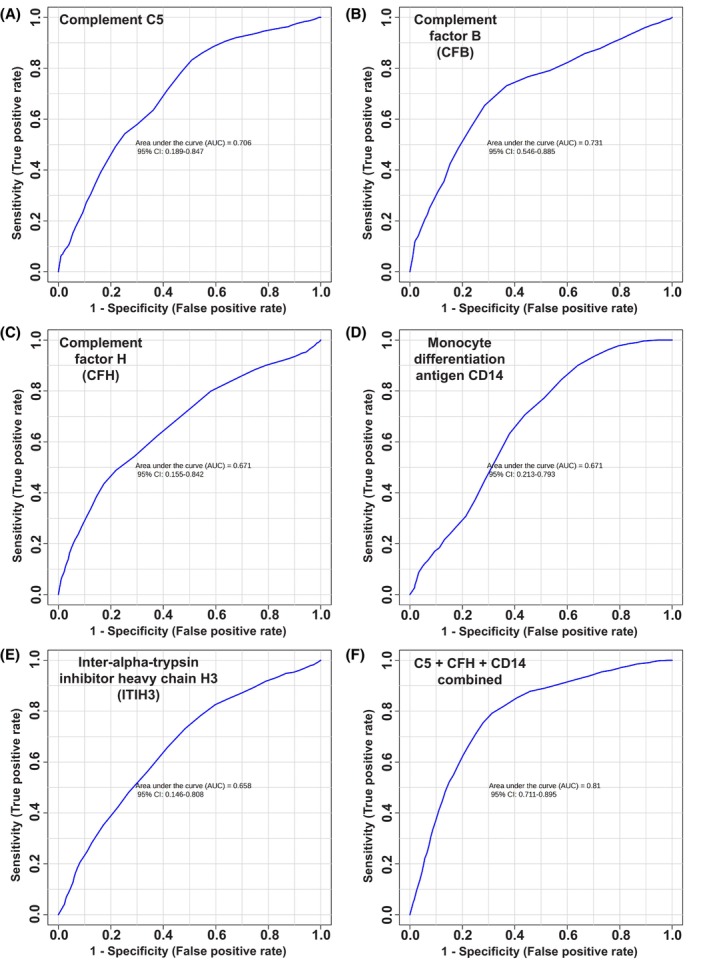
Human serum proteomics hits overlapping between KPC (*Kras* ^G12D/+^ *Trp53* ^R172H/+^ *Pdx1‐Cre*) mice and early‐stage PDAC patients, show promise in Receiver Operator Characteristic (ROC) curves, on metaboanalyst. ROC curves were generated using the early‐stage pancreatic ductal adenocarcinoma (PDAC) patient serum proteomics screen data (plotting 1‐sensitivity against sensitivity), (area under the curve (AUC) values and 95% confidence intervals (CIs) are shown on their respective ROC curves) shown for: (A) Complement C5 (C5) (AUC = 0.706), (B) Complement factor B (CFB) (AUC = 0.731), (C) Complement factor H (CFH) (AUC = 0.671), (D) Monocyte differentiation antigen CD14 (CD14) (AUC = 0.671), (E) Inter‐alpha‐trypsin inhibitor heavy chain H3 (ITIH3) (AUC = 0.658) and (F) combining C5, CFH and CD14 data (AUC = 0.810). Using the serum proteomics data of 62 benign symptomatic pancreaticobiliary disease case controls and 31 early‐stage PDAC patient samples.

Next, Receiver Operating Characteristic (ROC) plot analysis of the human serum proteomic screen data of the five hits [ITIH3, C5, CFB, CFH and CD14] that overlap between the KPC mice and early‐stage PDAC patients revealed that individually, the protein hits show promise in distinguishing early‐stage PDAC patients from benign symptomatic pancreaticobiliary disease cases (controls) (Fig. 7A–E), with area under the curves (AUCs) ranging from 0.658 to 0.731 [for ITIH3 and CFB, respectively]. However, subsequent analysis combining the proteins in pairs and then in triplicates revealed that the most optimal combination of proteins for distinguishing between the two groups of patients was C5, CFH and CD14, which gave an area under the curve (AUC) of 0.810 (Fig. 7F). This combination offered stronger predictive value than when additional proteins [CFB and/or ITIH3] were also combined into the signature (Fig. S5). Interestingly, when combining these signatures from our hits with the biomarker CA19‐9, the stronger combination [C5, CFH and CD14] showed an even higher predictive value, with an AUC of 0.856 compared to 0.814 without CA19‐9 (Fig. 7G,H).

Finally, to investigate therapeutic potential, we assessed the correlation between the expression of each of the five hits [ITIH3, C5, CFB, CFH and CD14] with the expression of KRAS and drug sensitivity to the KRAS inhibitor sotorasib, across cell lines categorised as ‘pancreatic adenocarcinoma’ for primary disease on DepMap. The results showed that only an increased expression of CFH significantly correlated with a lower sensitivity to sotorasib, whereas ITIH3 and C5 showed the opposite trend, where increased expression of each protein correlated with an increased sensitivity to sotorasib. However, unlike CFH, these correlations were not statistically significant. CD14 expression showed no correlation with sotorasib sensitivity (Fig. S6).

## 4 Discussion

In this study, we characterised the serum proteome of our two previously described GEMMs, which harbour abundant PanINs, and observed several hits consistent across both when compared to their wild‐type counterparts. Three proteins significantly decreased: alpha‐1‐antitrypsin 1–5, leukaemia inhibitory factor receptor and major urinary protein 17, and 5 proteins showed significant increases: ceruloplasmin, ITIH3, thyroxine‐binding globulin, E‐cadherin and tenascin. In the pre‐PDAC KPC mouse serum, we observed an even greater number of significant changes, with over 180 protein hits seen. Here, the significantly decreased hits were significantly enriched in proteins involved in carbon metabolism and peroxisome proliferator‐activated receptors (PPAR) signalling. Interestingly, PPAR signalling has recently been shown to be dysregulated in PDAC, with upregulated expression of the PPAR‐delta receptor seen in both mouse and human PanIN and PDAC tissue [37]. Another PPAR: PPAR‐gamma, has been shown to be capable of inducing pancreatic cancer cell proliferation, both *in vitro* and *in vivo*, by protecting against mitochondrial reactive oxygen species (ROS) [38]. However, to the best of our knowledge, our study on pre‐PDAC KPC mice is the first to identify dysregulated PPAR signalling in the serum. Conversely, the proteins that significantly increased in KPC serum were significantly enriched for proteins involved in complement and coagulation cascades, cholesterol metabolism and pancreatic secretion. Dysregulated cholesterol metabolism has already been linked to PDAC, with the cancer type shown to have a high cholesterol demand [39]. Complement cascades have also been linked to PDAC, with C3 and C3a levels in serum significantly higher in PDAC patients than controls [40]. Our data indicate that these pathways are also dysregulated and detectable in the serum of KPC mice, in the pre‐PDAC setting. As the data aligns with that of the literature, it gives confidence in the models and screens that were used in this project.

As PanINs are common in older humans and can also occur in noncancerous diseases, to reduce the possibility of detecting protein hits in the serum due to these PanINs, in our screen we used serum samples from age‐matched patients that had either early early‐stage PDAC or benign pancreaticobiliary disease. As such, we were able to filter out protein hits associated with age‐related PanINs that are not connected to early‐stage PDAC.

Our screen on early‐stage PDAC patient serum revealed that 25 proteins significantly change compared to benign pancreaticobiliary disease controls. The decreasing hits were significantly enriched in proteins involved in cholesterol metabolism and in processes linked to lipoproteins, whereas the increasing hits were enriched in proteins involved in the complement cascade as well as the immune response. Importantly, these enriched pathways that show dysregulation in patients are similar to those we observed in KPC mouse serum.

Assessment of overlap between the screens identified that five hits were common across the PanIN GEMMs and KPC mice [ceruloplasmin, E‐cadherin, ITIH3, tenascin and thyroxine‐binding globulin], with another five proteins common between the KPC mice and early‐stage PDAC patients [ITIH3, C5, CFB, CFH and CD14]. One hit, ITIH3, was common across all three screens. ITIH3 has previously been observed to be significantly elevated in the plasma of mice with PanIN‐3 lesions, by proteomics, and also in the serum from PDAC patients [41, 42]. Our study corroborates these findings while also identifying additional proteins associated with PanINs and early‐stage PDAC. In addition, our study also adds the novelty of the multi‐protein signatures that ITIH3 was identified in, as well as using serum samples solely from early‐stage (stage I‐II) PDAC patients.

The assessment of overlap also revealed that there were significant changes in proteins from both complement and coagulation pathways. Although not every model (PanIN‐abundant mice, KPC mice and PDAC human patients) showed differences in the same proteins from these pathways, all three models showed that these processes are dysregulated in both the mice and in PDAC patients.

For the eight murine hits (in the PanIN GEMMs), only three were seen in the early‐stage PDAC patient serum proteomics screen and only one [ITIH3] reached statistical significance. The other five hits in the PanIN GEMMs were not seen at all in the human screen data. This could partly explain the poor overlap between the PanIN GEMMs and the early‐stage PDAC patients.

For the five serum proteomics hits overlapping between the KPC mice and early‐stage PDAC patients [ITIH3, C5, CFB, CFH and CD14], we observed a positive correlation between the stage of PDAC and the levels of the protein of interest, using a publicly available tissue proteomics dataset; increasing from normal tissue to PDAC and through the progressive PDAC stages (1–4) assessed. This increase was significant for most of the comparisons against normal tissue, suggesting that these proteins are involved in PDAC initiation. However, it may also be that these proteins are associated with PDAC development or are a consequence of PDAC development and are not drivers. In further work that assessed for an association between these five protein hits using tissue proteomic data on cbioportal, we observed that all five significantly and positively associate with each other. This implies that these proteins could be controlled by a common mechanism or are perhaps involved in the same process in terms of PDAC initiation.

Through ROC curve analysis, we observed that individually the serum proteomics hits show a degree of promise for distinguishing between the early‐stage PDAC patients and the benign pancreaticobiliary disease case controls. However, the real promise comes from combining a subset of the protein hits into a biomarker. An AUC of 0.810 in a ROC curve was achieved when data from C5, CFH and CD14 were combined. This suggests that this trio of proteins has the greatest promise in constituting a signature to distinguish those with early‐stage PDAC from benign controls.

CD14 has previously been linked to PDAC, where a study showed an increase in plasma CD14 levels in diabetic patients with PDAC, compared to diabetic controls [43]. This supports the results of our study, suggesting that CD14 may have a role in PDAC development. However, here we show a signature identified in KPC before PDAC development, which translates to human patients, indicating that it may be useful in detecting PDAC at the very early stages of disease. Moreover, a recent study identified a new cancer‐associated fibroblast (CAF) subset in human PDAC, termed complement‐secreting CAFs (cs‐CAFs), which by single‐cell RNA‐sequencing showed elevated transcription of genes involved in the complement system, including C3, C7, CFB, CFD, CFH and CFI, compared with the other CAFs assessed [44]. Perhaps the complement system proteins identified in our serum proteomic screens reflect the potential presence of cs‐CAFs in the KPC mice and early‐stage PDAC patients.

## 5 Conclusions

Our findings have identified serum proteomics hits, in both GEMMs with abundant PanINs and KPC mice in the pre‐PDAC state, as well as identifying partial overlap between changes in the mice and those identified in early‐stage PDAC patients. The protein trio identified in this study (C5, CFH and CD14) can distinguish between those with early‐stage PDAC and benign pancreaticobiliary disease cases with a high degree of confidence. Further studies are crucial for validating our findings in additional patient cohorts, with the goal of identifying markers of early‐stage PDAC and PanINs in high‐risk individuals.

## Conflict of interest

Kevin M. Ryan is the Editor‐in‐Chief of *Molecular Oncology*, but has taken no role in, nor was he party to, any stage of the peer review of the manuscript. Kevin M. Ryan and Hannah Mearns are the co‐inventors on a filed patent (application number GB2517669.4) relating to the biomarkers identified in this study and described in the manuscript. The authors declare no further conflicts of interest.

## Author contributions

KMR, PA, SPP and SZ conceived the study. HM, JSL, SL, KH, MJGWL and PFP designed, conducted and analysed experiments. CN conducted experiments. HM and KMR wrote the manuscript.

## Supporting information


**Fig. S1.** PanIN GEMMs histology, AB/PAS staining and PCA plot.

**Fig. S2.** KPC mouse serum proteomics PCA plot and Ki67 staining.

**Fig. S3.** Early‐stage human PDAC patient serum proteomics PCA plot, heatmap of a publicly available dataset of human PDAC proteomics and serum CA19‐9 levels.

**Fig. S4.** Immunohistochemistry (IHC) staining of pre‐PDAC KPC pancreas tissue for the hits ITIH3, CD14, CFB and CFH, as well as for α‐SMA and vimentin as cancer‐associated fibroblast (CAF) markers.

**Fig. S5.** Additional Receiver Operator Characteristic (ROC) curves of pairwise and multiple protein combinations of hits from the early‐stage PDAC patient serum proteomics screen data, on metaboanalyst.

**Fig. S6.** Correlation analysis of gene expression of the 5 hits with the drug sensitivity to the KRAS inhibitor sotorasib in DepMap database.

**Table S1.** Genotypes in the PanIN GEMM screen.
**Table S2.** Serum proteomics hits in the ATG7 PanIN GEMMs (*Pdx1‐Cre Atg7* ^fl/fl^ *Kras* ^G12D/+^).
**Table S3.** Serum proteomics hits in the ATG5 PanIN GEMMs (*Pdx1‐Cre Atg5* ^fl/fl^ *Kras* ^G12D/+^).
**Table S4.** Serum proteomics hits in the KPC (*Kras* ^G12D/+^ *Trp53* ^R172H/+^ *Pdx1‐Cre*) mice.
**Table S5.** Serum proteomics hits in the early‐stage PDAC patients.

## Data Availability

The raw files and the spectronaut search results files have been deposited to the ProteomeXchange Consortium via the PRIDE partner repository in three parts (PanIN mouse model data, KPC mouse data and human PDAC patient data), with the dataset identifiers: PXD065578, PXD065698 and PXD065581, respectively [45, 46].
