# Supplementary figures and images for: Identification of serum protein biomarkers for pre‐cancerous lesions associated with pancreatic ductal adenocarcinoma
Source: Mol Oncol. 2026 Feb 18;20(6):1473–93. doi: 10.1002/1878-0261.70213 (PMC13238842; doi:10.1002/1878-0261.70213)

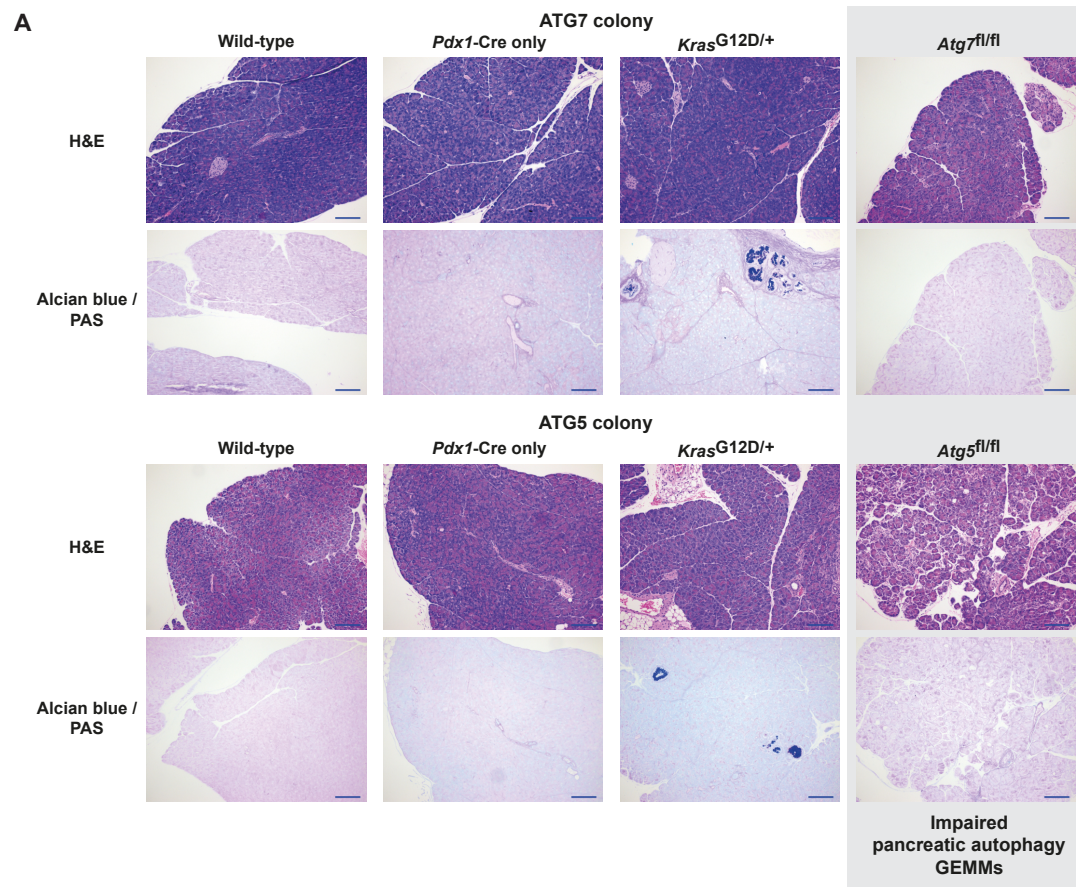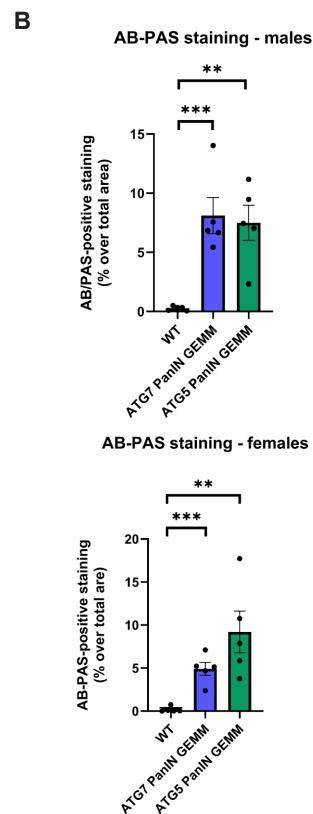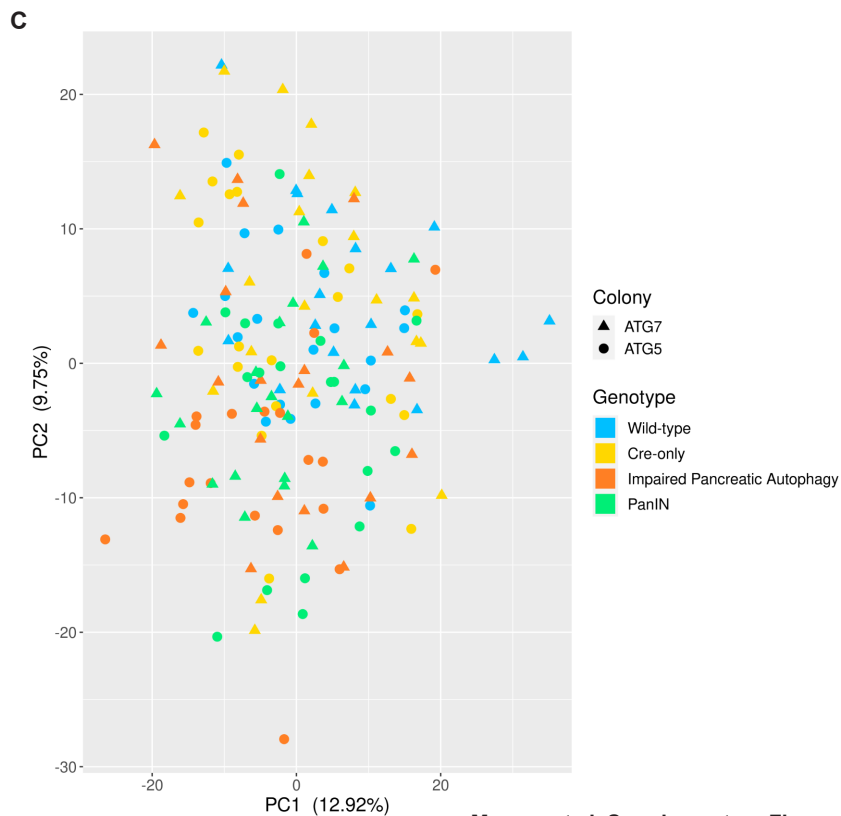

Supplement: Supplementary file 1 — Fig. S1. PanIN GEMMs histology, AB/PAS staining and PCA plot. [file MOL2-20-1473-s006.pdf]

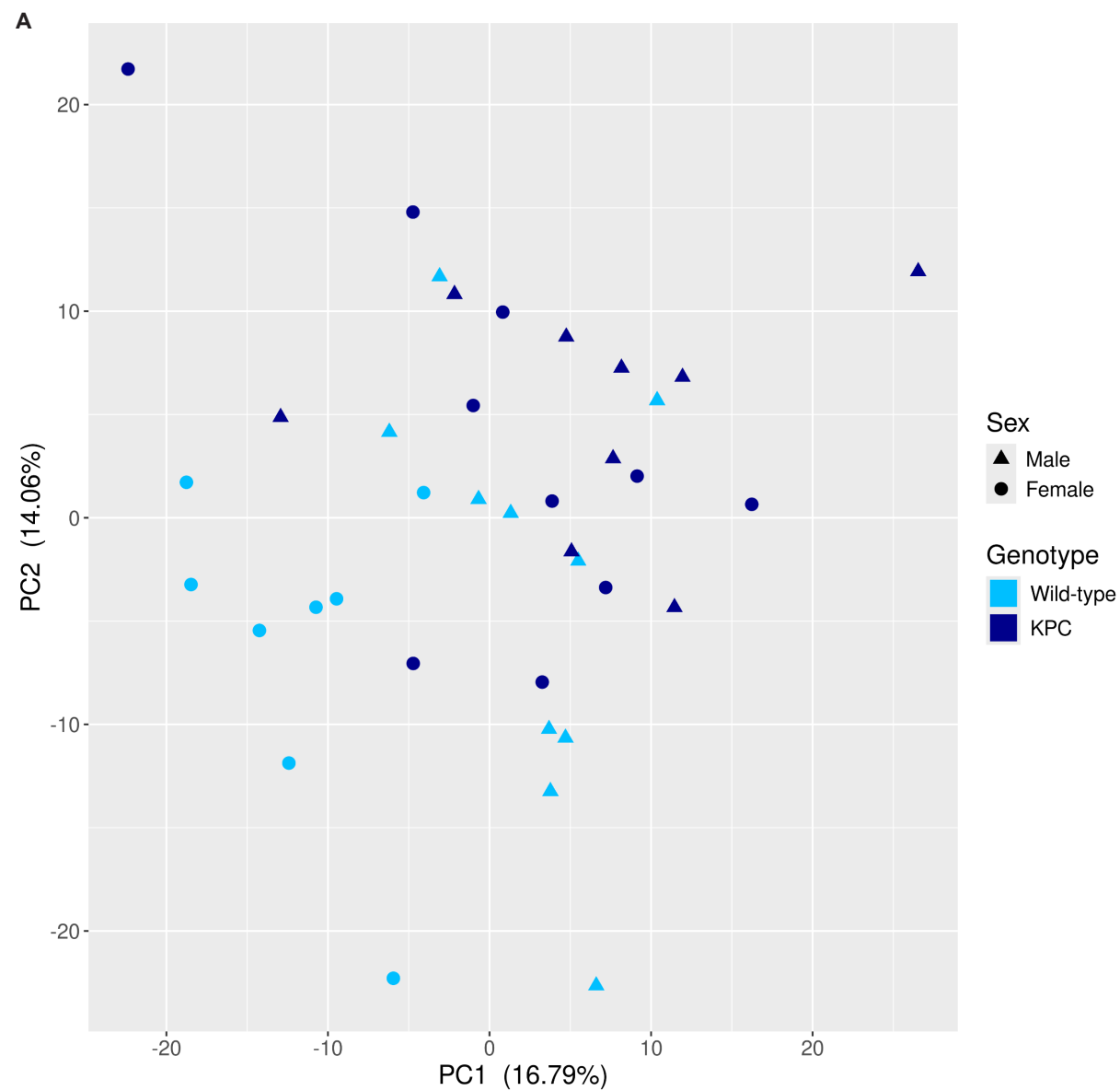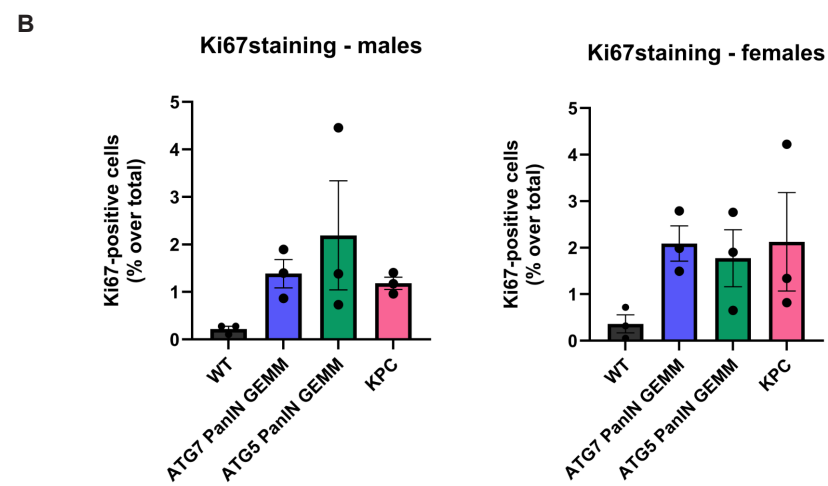

Supplement: Supplementary file 2 — Fig. S2. KPC mouse serum proteomics PCA plot and Ki67 staining. [file MOL2-20-1473-s004.pdf]

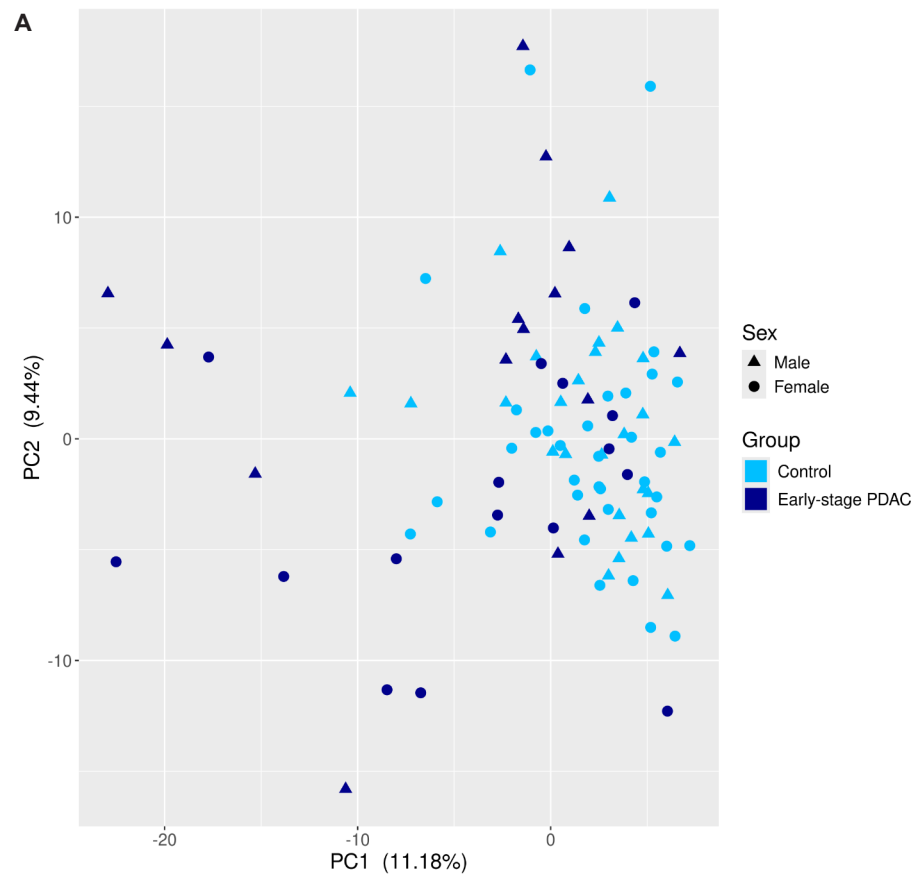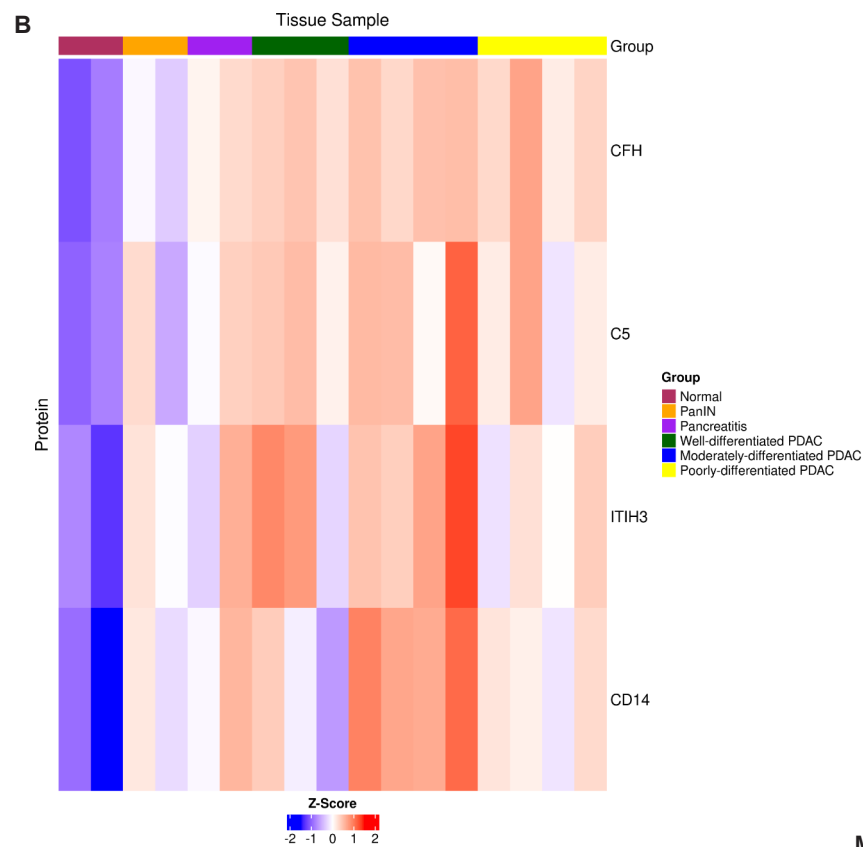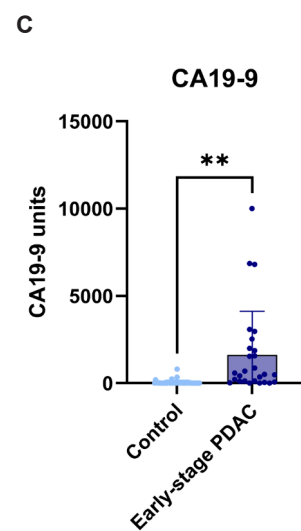

Supplement: Supplementary file 3 — Fig. S3. Early‐stage human PDAC patient serum proteomics PCA plot, heatmap of a publicly available dataset of human PDAC proteomics and serum CA19‐9 levels. [file MOL2-20-1473-s002.pdf]

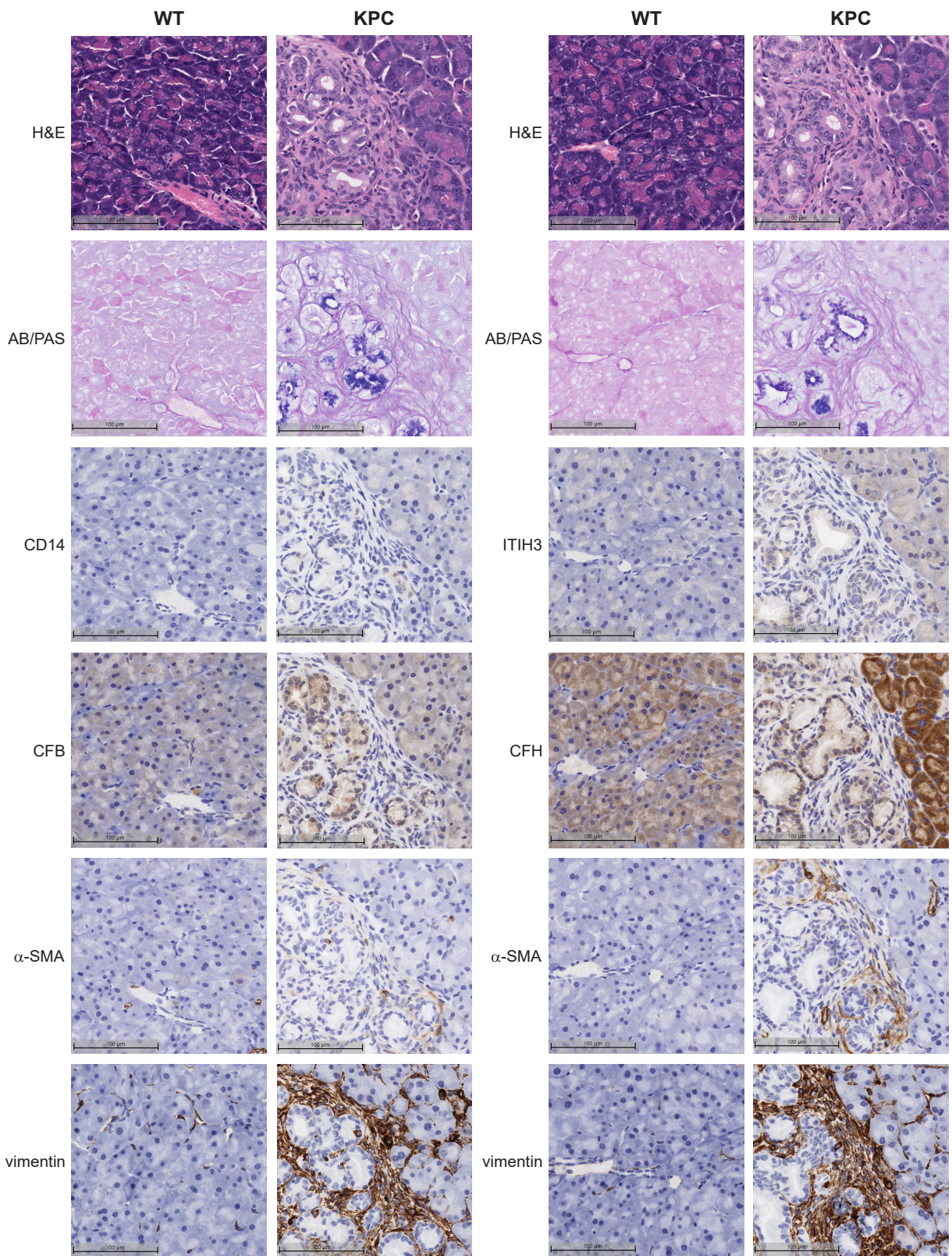

Supplement: Supplementary file 4 — Fig. S4. Immunohistochemistry (IHC) staining of pre‐PDAC KPC pancreas tissue for the hits ITIH3, CD14, CFB and CFH, as well as for α‐SMA and vimentin as cancer‐associated fibroblast (CAF) markers. [file MOL2-20-1473-s003.pdf]

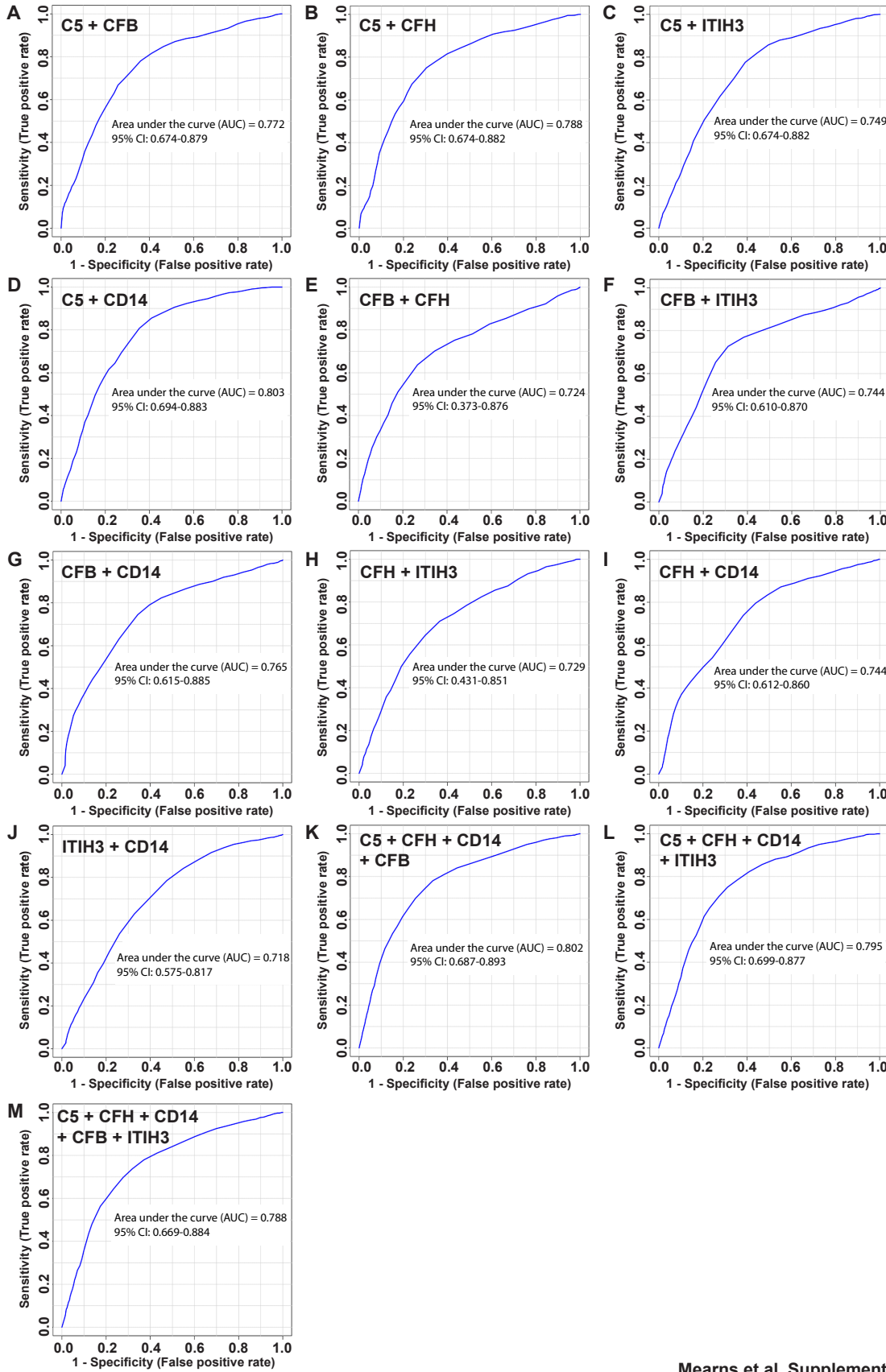

Supplement: Supplementary file 5 — Fig. S5. Additional Receiver Operator Characteristic (ROC) curves of pairwise and multiple protein combinations of hits from the early‐stage PDAC patient serum proteomics screen data, on metaboanalyst. [file MOL2-20-1473-s007.pdf]

**A****ITIH3**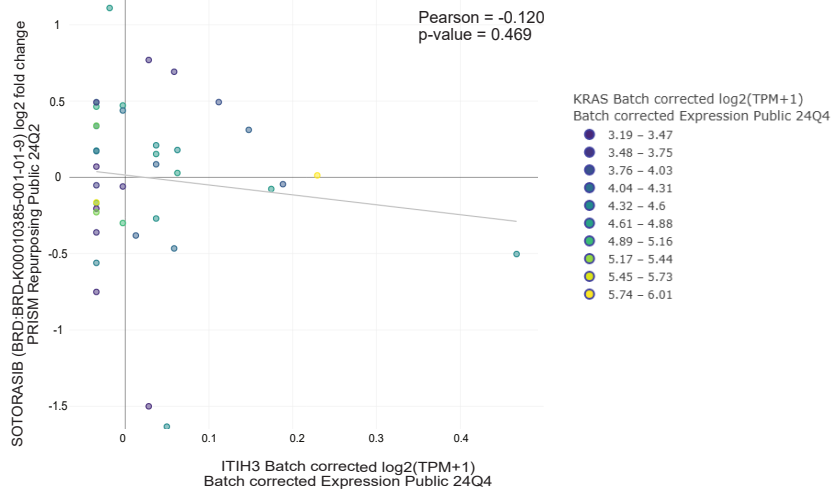**B****C5**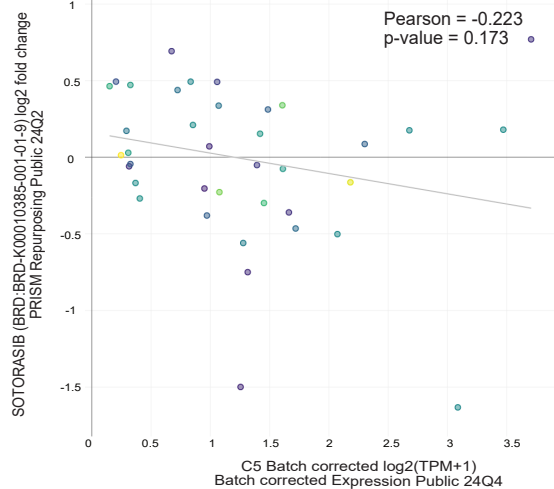**C****CFB**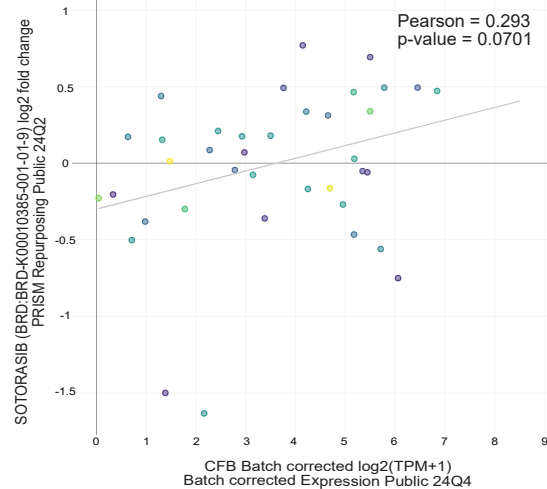**D****CFH**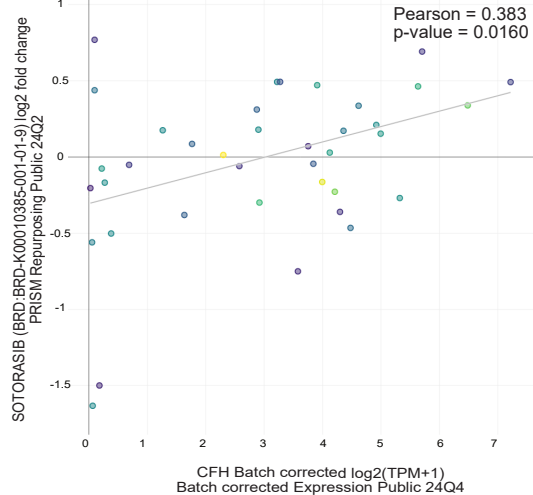**E****CD14**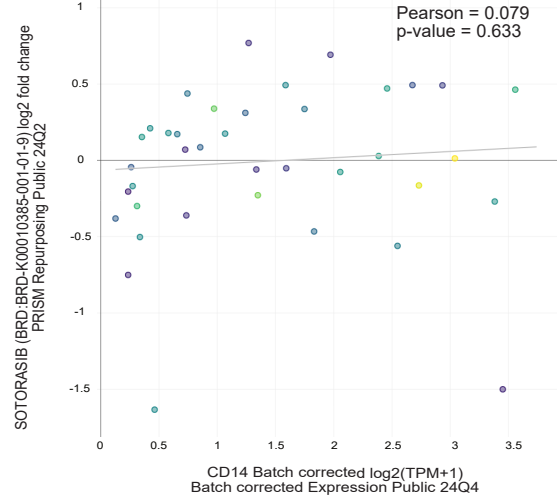

Supplement: Supplementary file 6 — Fig. S6. Correlation analysis of gene expression of the 5 hits with the drug sensitivity to the KRAS inhibitor sotorasib in DepMap database. [file MOL2-20-1473-s001.pdf]
