# Supplementary material for: Identification of serum protein biomarkers for pre‐cancerous lesions associated with pancreatic ductal adenocarcinoma
Source: Mol Oncol. 2026 Feb 18;20(6):1473–93. doi: 10.1002/1878-0261.70213 (PMC13238842; doi:10.1002/1878-0261.70213)
Supplement: Supplementary file 7 — Table S1. Genotypes in the PanIN GEMM screen. Table S2. Serum proteomics hits in the ATG7 PanIN GEMMs (Pdx1‐Cre Atg7 fl/fl Kras G12D/+). Table S3. Serum proteomics hits in the ATG5 PanIN GEMMs (Pdx1‐Cre Atg5 fl/fl Kras G12D/+). Table S4. Serum proteomics hits in the KPC (Kras G12D/+ Trp53 R172H/+ Pdx1‐Cre) mice. Table S5. Serum proteomics hits in the early‐stage PDAC patients. [file MOL2-20-1473-s005.pdf]

Supplementary tables and supplementary figure legends

Supplementary Tables

	Group	Genotype	ATG colony
**1**	Wild-type	-	ATG7
**2**	Cre-only	*Pdx1-Cre*	ATG7
**3**	Impaired pancreatic autophagy	*Atg7*$^{fl/fl}$ *Pdx1-Cre*	ATG7
**4**	PanIN GEMM	Lox-STOP-Lox-*Kras*$^{G12D/+}$ *Atg7*$^{fl/fl}$ *Pdx1-Cre*	ATG7
**5**	Wild-type	-	ATG5
**6**	Cre-only	*Pdx1-Cre*	ATG5
**7**	Impaired pancreatic autophagy	*Atg5*$^{fl/fl}$ *Pdx1-Cre*	ATG5
**8**	PanIN GEMM	Lox-STOP-Lox-*Kras*$^{G12D/+}$ *Atg5*$^{fl/fl}$ *Pdx1-Cre*	ATG5

Supplementary Table 1. Genotypes in the PanIN GEMM screen. 8 groups of mice used in the ONE-way analysis of variance (ANOVA) in the analysis of the pancreatic intraepithelial neoplasm (PanIN) genetically engineered mouse model (GEMM) serum proteomics screens. Group, genotype and ATG colony (either ATG7 or ATG5) are shown.

Gene symbol	Increasing Protein Hits	Log2(fold-change) (between ATG7 PanIN GEMMs and wild-type mice)	Adjusted p-value
Albfm1	Albumin superfamily member 1	0.24	5.18×10^{-4}
Amy1	Alpha-amylase 1	0.40	2.34×10^{-3}
Man2a1	Alpha-mannosidase 2	0.45	0
Anpep	Aminopeptidase N	0.53	0
B4galnt1	Beta-1,4 N-acetylgalactosaminyltransferase 1	0.60	0
Hexa	Beta-hexosaminidase subunit alpha	0.51	1.68×10^{-4}
Cdh1	Cadherin-1	0.89	0
Cp	Ceruloplasmin	0.25	0
F12	Coagulation factor XII	0.36	2.24×10^{-3}
Colec10	Collectin-10	0.57	8.00×10^{-5}
SWISS-PROT:Q2UVX4	Complement C3 precursor ((Bos taurus)	0.42	4.60×10^{-3}
Dsg2	Desmoglein-2	0.53	7.92×10^{-5}
Erap1	Endoplasmic reticulum aminopeptidase 1	0.45	0
Hspa5	Endoplasmic reticulum chaperone BiP	0.37	3.13×10^{-2}
Ecm1	Extracellular matrix protein 1	0.65	1.23×10^{-3}
Fetub	Fetuin-B	0.52	0
Fn1	Fibronectin	0.30	2.04×10^{-2}
Ggh	Gamma-glutamyl hydrolase;Isoform II of Gamma-glutamyl hydrolase	0.42	4.64×10^{-4}
H6pd	GDH/6PGL endoplasmic bifunctional protein	0.81	0
Kng2	HMW kininogen-II	0.30	4.28×10^{-3}
	Ig heavy chain V region M603	1.10	2.27×10^{-3}
Ighv7-1	Immunoglobulin heavy variable 7-1 (Fragment)	0.98	2.29×10^{-3}
Itih3	Inter-alpha-trypsin inhibitor heavy chain H3	0.43	0
Tnc	Isoform 2 of Tenascin	0.55	0
Qsox1	Isoform 3 of Sulfhydryl oxidase 1	1.08	1.22×10^{-2}
Mrc1	Macrophage mannose receptor 1	0.36	0
Ncam1	Neural cell adhesion molecule 1;Isoform 2 of Neural cell adhesion molecule 1;Isoform 3 of Neural cell adhesion molecule 1	0.40	1.39×10^{-4}
Pla2g7	Platelet-activating factor acetylhydrolase	0.52	1.38×10^{-3}
Galnt2	Polypeptide N-acetylgalactosaminyltransferase 2; Isoform 2 of Polypeptide N-acetylgalactosaminyltransferase 2	0.70	4.43×10^{-2}
Qsox1	Sulfhydryl oxidase 1	0.31	0
Serpina7	Thyroxine-binding globulin	1.03	0
Tfrc	Transferrin receptor protein 1	0.59	1.38×10^{-4}
Mmp2	type IV collagenase (72 kDa)	0.39	1.40×10^{-2}
Pros1	Vitamin K-dependent protein S	0.24	0

Gene Symbol	Decreasing Protein Hits	Log2(fold-change) (between ATG7 PanIN GEMMs and wild-type mice)	Adjusted p-value
Adipoq	Adiponectin	-0.74	4.27×10^{-2}

Hba-a1	Alpha globin 1	-0.93	2.68 x 10^{-3}
Orm1	Alpha-1-acid glycoprotein 1	-0.45	1.38 x 10^{-3}
Serpina1e	Alpha-1-antitrypsin 1-5	-2.40	7.48 x 10^{-5}
Ctrb1	Chymotrypsinogen B	-1.89	0
C1ra	Complement C1r-A subcomponent	-0.37	1.23 x 10^{-3}
C1s1	Complement component 1, s subcomponent 1	-0.78	9.68 x 10^{-3}
C8g	Complement component C8 gamma chain	-1.22	1.22 x 10^{-3}
Ckm	Creatine kinase M-type	-1.94	1.58 x 10^{-2}
Clec11a	C-type lectin domain family 11 member A	-0.48	1.75 x 10^{-3}
Alad	Delta-aminolevulinic acid dehydratase	-1.17	3.82 x 10^{-2}
Ftl1	Ferritin	-1.00	0
Fmn2	Formin-2	-0.93	3.82 x 10^{-4}
Gapdh	Glyceraldehyde-3-phosphate dehydrogenase	-1.26	4.87 x 10^{-2}
Mstn	Growth/differentiation factor 8	-0.61	3.73 x 10^{-3}
Ighv1-47	Immunoglobulin heavy variable 1-47	-1.72	0
Igf1	Insulin-like growth factor I (Fragment);Insulin-like growth factor I;Isoform IGF-IB of Insulin-like growth factor I	-0.78	1.40 x 10^{-3}
Ghr	Isoform 2 of Growth hormone receptor	-0.63	5.56 x 10^{-4}
Il1rap	Isoform 2 of Interleukin-1 receptor accessory protein	-0.33	8.81 x 10^{-3}
Lifr	Leukemia inhibitory factor receptor	-0.77	0
Mup1	Major urinary protein 1	-2.93	0
Mup17	Major urinary protein 17	-3.30	0
Mup2	Major urinary protein 2	-2.45	0
Mug1	Murinoglobulin-1	-0.67	2.22 x 10^{-4}
Mb	Myoglobin	-1.97	2.94 x 10^{-2}
Ppia	Peptidyl-prolyl cis-trans isomerase A	-0.91	2.95 x 10^{-2}
Ppic	Peptidyl-prolyl cis-trans isomerase C	-0.42	1.24 x 10^{-2}
Gm8797;Kxd1;Ubc;Uba52;Kxd1;Ubb;Ubc;Rps27a;Uba52;Ubb	Predicted pseudogene 8797;KxDL motif-containing protein 1 (Fragment);Polyubiquitin-C (Fragment);Ubiquitin-60S ribosomal protein L40 (Fragment);Ubiquitin-60S ribosomal protein L40;Polyubiquitin-B;Polyubiquitin-C;Ubiquitin-40S ribosomal protein S27a;Ubiquitin-60S ribosomal protein L40;Polyubiquitin-B (Fragment)	-0.64	2.72 x 10^{-3}
Serpina11	Serpin A11	-1.35	0
Sod1	Superoxide dismutase [Cu-Zn]	-1.79	7.83 x 10^{-3}

Supplementary Table 2. Serum proteomics hits in the ATG7 PanIN GEMMs (*Pdx1-Cre Atg7*$^{fl/fl}$ *Kras*$^{G12D/+}$). Protein hits identified in the serum proteomics screen, in the ATG7 pancreatic intraepithelial neoplasm (PanIN) genetically engineered mouse models (GEMMs) at 90-days (+/- 10 days) old. 34 significantly increasing hits and 30 significantly decreasing hits. The gene name, protein name, log2(fold-change) and adjusted p-values for each hit are shown.

Gene symbol	Increasing Protein Hit	Log2(fold-change) (between ATG5 PanIN GEMMs and wild-type mice)	Adjusted p-value
Cdh1	Cadherin-1	0.72	0
Cdh5	Cadherin-5	0.43	1.24×10^{-3}
Cp	Ceruloplasmin	0.23	0
Fcgbp	Fc fragment of IgG-binding protein	0.73	1.25×10^{-3}
Hp	Haptoglobin	2.78	7.47×10^{-4}
Itih3	Inter-alpha-trypsin inhibitor heavy chain H3	0.38	0
Tnc	Isoform 2 of Tenascin	0.53	0
Vnn1	Pantetheinase	0.75	0
Serpina7	Thyroxine-binding globulin	1.21	0
**Gene symbol**	**Decreasing Protein Hit**	**Log2(fold-change) (between ATG5 PanIN GEMMs and wild-type mice)**	**Adjusted p-value**
Serpina1d	Alpha-1-antitrypsin 1-4	-0.79	1.23×10^{-3}
Serpina1e	Alpha-1-antitrypsin 1-5	-2.46	7.48×10^{-5}
Apoc1	Apolipoprotein C-I	-0.51	1.79×10^{-2}
Apoc3	Apolipoprotein C-III	-0.72	0
Apoc4	Apolipoprotein C-IV	-0.92	0
Apon	Apolipoprotein N	-0.64	7.51×10^{-3}
Cela1	Chymotrypsin-like elastase family member 1	-1.71	0
F7	Coagulation factor VII	-0.46	1.15×10^{-3}
Cfhr1	Complement factor H-related 1	-0.31	0
Igfbp3	Insulin-like growth factor-binding protein 3	-0.65	0
Igfals	Insulin-like growth factor-binding protein complex acid labile subunit	-0.51	2.44×10^{-3}
Lifr	Leukemia inhibitory factor receptor	-0.71	0
Mup17	Major urinary protein 17	-2.31	0
Serpina3k	Serine protease inhibitor A3K	-2.04	3.28×10^{-3}

Supplementary Table 3. Serum proteomics hits in the ATG5 PanIN GEMMs (*Pdx1-Cre Atg5*$^{fl/fl}$ *Kras*$^{G12D/+}$). Protein hits identified in the serum proteomics screen, in the ATG5 PanIN GEMMs at 90-days (+/- 10 days) old. 9 significantly increasing hits and 14 significantly decreasing hits. The gene name, protein name, log2(fold-change) and adjusted p-values for each hit are shown.

Gene symbol	Increasing Protein Hit	Log2(fold-change) (between KPC and wild-type)	Adjusted p-value
Adgre5	Adhesion G protein-coupled receptor E5	0.75	0
Adh1	Alcohol dehydrogenase 1	1.83	2.50×10^{-2}
Orm1	Alpha-1-acid glycoprotein 1	0.93	0
Orm2	Alpha-1-acid glycoprotein 2	1.44	0
Serpinf2	Alpha-2-antiplasmin	0.53	0
Ahsg	Alpha-2-HS-glycoprotein	0.48	1.05×10^{-3}

Amy1	Alpha-amylase 1	0.57	7.26×10^{-3}
Angptl3	Angiopoietin-related protein 3	0.69	5.16×10^{-4}
Agt	Angiotensinogen	0.34	1.18×10^{-2}
Slpi	Antileukoproteinase	0.45	4.85×10^{-2}
Apoa1	Apolipoprotein A-I	0.39	3.39×10^{-2}
Apoa4	Apolipoprotein A-IV	0.48	1.60×10^{-2}
Apoa5	Apolipoprotein A-V	0.89	1.48×10^{-4}
Apob	Apolipoprotein B-100	0.51	8.77×10^{-4}
Apoe	Apolipoprotein E	0.44	2.66×10^{-2}
Apom	Apolipoprotein M	0.41	1.24×10^{-2}
Apon	Apolipoprotein N	0.71	3.59×10^{-3}
Apoh	Beta-2-glycoprotein 1	0.41	3.51×10^{-3}
C4bpa	C4b-binding protein	0.69	9.73×10^{-4}
Cdh1	Cadherin-1	0.82	0
Cpb2	Carboxypeptidase B2	0.64	0
Ctsa	Carboxypeptidase;Lysosomal protective protein	0.39	8.75×10^{-3}
Camp	Cathelicidin antimicrobial peptide	1.38	0
Ctsb	Cathepsin B	0.8	0
Ctsd	Cathepsin D	0.39	1.25×10^{-2}
Ctse	Cathepsin E	0.56	2.59×10^{-2}
Ctss	Cathepsin S	0.67	3.28×10^{-4}
Ctsz	Cathepsin Z	0.44	2.19×10^{-3}
Ccl8	C-C motif chemokine 8	0.93	7.03×10^{-3}
Cd9	CD9 antigen	0.85	3.78×10^{-2}
Cp	Ceruloplasmin	0.8	0
Cela2a	Chymotrypsin-like elastase family member 2A	0.85	2.96×10^{-2}
Ctrb1	Chymotrypsinogen B	2.06	0
F9	Coagulation factor IX	0.75	0
F5	Coagulation factor V	0.58	0
F7	Coagulation factor VII	0.98	0
F10	Coagulation factor X	0.43	1.07×10^{-2}
Col18a1	Collagen alpha-1(XVIII) chain; Isoform 2 of Collagen alpha-1(XVIII) chain; Isoform 3 of Collagen alpha-1(XVIII) chain	0.65	0
Colec10	Collectin-10	1.51	5.71×10^{-4}
C1qa	Complement C1q subcomponent subunit A	0.64	3.76×10^{-3}
C1qb	Complement C1q subcomponent subunit B	0.65	3.96×10^{-3}
C1qc	Complement C1q subcomponent subunit C	0.55	7.16×10^{-3}
C1ra	Complement C1r-A subcomponent	0.43	6.60×10^{-3}
C1s1	Complement C1s-1 subcomponent	0.51	6.06×10^{-4}
C3	Complement C3	0.77	0
C4b	Complement C4-B	1.26	0
C5	Complement C5	0.67	7.53×10^{-3}

C1s1	Complement component 1; s subcomponent 1	1.05	7.89 x 10^{-3}
Gm20547	Complement factor B	0.66	0
Cfh	Complement factor H	0.79	0
Cfi	Complement factor I	0.67	0
Clec4f	C-type lectin domain family 4 member F	1.25	0
Dsg2	Desmoglein-2	0.41	1.86 x 10^{-2}
Ctbs	Di-N-acetylchitobiase	0.53	6.25 x 10^{-4}
Hspa5	Endoplasmic reticulum chaperone BiP	0.43	1.20 x 10^{-3}
AI182371	Expressed sequence AI182371	0.59	0
Fetub	Fetuin-B	0.57	0
Fgl1	Fibrinogen-like protein 1	1.74	0
Fn1	Fibronectin	0.75	0
Fcn1	Ficolin-1	0.74	0
Fstl1	Follistatin-related protein 1	0.39	4.47 x 10^{-2}
Aldob	Fructose-bisphosphate aldolase B	0.74	4.62 x 10^{-2}
Ggh	Gamma-glutamyl hydrolase; Isoform II of Gamma-glutamyl hydrolase	0.38	2.25 x 10^{-2}
H6pd	GDH/6PGL endoplasmic bifunctional protein	0.86	0
Gnmt	Glycine N-methyltransferase	1.29	2.74 x 10^{-2}
Pygl	Glycogen phosphorylase, liver form	1.1	7.19 x 10^{-3}
H2-L	H-2 class I histocompatibility antigen, L-D alpha chain	0.57	1.27 x 10^{-3}
H2-Q10	H-2 class I histocompatibility antigen, Q10 alpha chain	0.45	4.15 x 10^{-3}
H2-Q8	H-2 class I histocompatibility antigen, Q8 alpha chain	0.55	3.92 x 10^{-3}
Hp	Haptoglobin	5.16	0
Hpx	Hemopexin	0.84	0
Serpind1	Heparin cofactor 2	0.34	1.40 x 10^{-2}
Mst1	Hepatocyte growth factor-like protein	0.51	1.08 x 10^{-3}
H2-Q1	Histocompatibility 2, Q region locus 1	0.61	1.64 x 10^{-2}
H2-Q4	Histocompatibility 2, Q region locus 4	0.45	2.14 x 10^{-4}
Habp2	Hyaluronan-binding protein 2; Isoform 2 of Hyaluronan-binding protein 2	0.45	1.40 x 10^{-3}
Ighg1	Ig gamma-1 chain C region secreted form	0.72	9.76 x 10^{-3}
Ighv1-37	Immunoglobulin heavy variable 1-37; Immunoglobulin heavy variable 1-37 (Fragment)	1.07	9.05 x 10^{-3}
Ighv14-4	Immunoglobulin heavy variable 14-4; Immunoglobulin heavy variable 14-4 (Fragment)	1.57	3.68 x 10^{-3}
Ighv1-62-1	Immunoglobulin heavy variable 1-62-1	1.06	1.38 x 10^{-2}
Ighv2-9	Immunoglobulin heavy variable 2-9 (Fragment)	0.93	2.68 x 10^{-2}
Ighv5-9	Immunoglobulin heavy variable 5-9 (Fragment)	1.95	2.22 x 10^{-3}
Igkv6-13	Immunoglobulin kappa variable 6-13	0.67	2.91 x 10^{-2}

Igfbp3	Insulin-like growth factor-binding protein 3	0.32	3.42×10^{-2}
Itih4	Inter alpha-trypsin inhibitor, heavy chain 4	1.3	0
Itih4	Inter alpha-trypsin inhibitor, heavy chain 4	1.56	0
Itih1	Inter-alpha-trypsin inhibitor heavy chain H1	0.52	3.33×10^{-4}
Itih3	Inter-alpha-trypsin inhibitor heavy chain H3	1.39	0
Icam1	Intercellular adhesion molecule 1; Isoform 2 of Intercellular adhesion molecule 1	0.32	2.90×10^{-2}
Tnc	Isoform 2 of Tenascin	0.56	0
Ifnar2	Isoform 3 of Interferon alpha/beta receptor 2	0.39	2.17×10^{-3}
Qsox1	Isoform 3 of Sulfhydryl oxidase 1	1.55	2.20×10^{-3}
Lrg1	Leucine-rich HEV glycoprotein	1.38	0
Lbp	Lipopolysaccharide-binding protein	1.04	0
Sell	L-selectin	0.39	3.26×10^{-2}
Loxl1	Lysyl oxidase homolog; Lysyl oxidase homolog 1	0.36	4.08×10^{-2}
Csf1r	Macrophage colony-stimulating factor 1 receptor	0.8	1.33×10^{-3}
Mrc1	Macrophage mannose receptor 1	0.32	2.69×10^{-2}
Mup2	Major urinary protein 2	0.92	1.67×10^{-2}
Mbl1	Mannose-binding protein A	0.38	1.86×10^{-2}
Cd14	Monocyte differentiation antigen CD14	0.88	9.63×10^{-3}
Minpp1	Multiple inositol polyphosphate phosphatase 1	0.49	1.51×10^{-4}
Pglyrp2	N-acetylmuramoyl-L-alanine amidase	0.4	2.69×10^{-3}
Napsa	Napsin-A	0.39	1.17×10^{-2}
Oaf	Out at first protein homolog	0.45	1.04×10^{-2}
Lcat	Phosphatidylcholine-sterol acyltransferase	0.51	6.96×10^{-4}
Pltp	Phospholipid transfer protein	0.64	8.00×10^{-5}
Serpinf1	Pigment epithelium-derived factor	0.59	0
Serping1	Plasma protease C1 inhibitor	0.58	0
Plg	Plasminogen	0.48	0
Pla2g7	Platelet-activating factor acetylhydrolase	0.51	8.89×10^{-4}
Pcyox1	Prenylcysteine oxidase 1	0.56	0
Grn	Progranulin	0.69	3.45×10^{-4}
Cfp	Properdin	0.49	2.26×10^{-2}
Pcsk9	Proprotein convertase subtilisin/kexin type 9	0.92	1.71×10^{-2}
Psap	Prosaposin	0.71	0
Ambp	Protein AMBP	0.9	0
S100a9	Protein S100-A9	1.37	8.00×10^{-4}

Serpina10	Protein Z-dependent protease inhibitor	0.9	0
Prg4	Proteoglycan 4	1.56	0
Reg3b	Regenerating islet-derived protein 3-beta	1.2	6.57×10^{-4}
Retnla	Resistin-like alpha	0.82	1.16×10^{-3}
Rnase4	Ribonuclease 4	0.67	7.84×10^{-5}
Selenop	Selenoprotein P	0.47	3.39×10^{-4}
Serpina3m	Serine protease inhibitor A3M	0.35	2.71×10^{-2}
Serpina3n	Serine protease inhibitor A3N	0.92	0
Serpina3n	Serine protease inhibitor A3N	0.94	2.11×10^{-4}
Trf	Serotransferrin	0.39	1.09×10^{-3}
Saa4	Serum amyloid A-4 protein	0.9	1.25×10^{-3}
Apcs	Serum amyloid P-component	3.85	0
Qsox1	Sulfhydryl oxidase 1	0.72	0
Serpina7	Thyroxine-binding globulin	1.23	7.69×10^{-5}
Fuca1	Tissue alpha-L-fucosidase	1.27	4.53×10^{-3}
Vcam1	Vascular cell adhesion protein 1	0.55	0
Gc	Vitamin D-binding protein	0.43	1.41×10^{-3}
Proc	Vitamin K-dependent protein C	0.64	6.47×10^{-4}
Pros1	Vitamin K-dependent protein S	0.37	1.18×10^{-2}
Proz	Vitamin K-dependent protein Z	0.91	0
Vtn	Vitronectin	0.66	0

**Gene symbol**	**Decreasing Protein Hit**	**Log2(fold-change) (between KPC and wild-type)**	**Adjusted p-value**
Slc3a2	4F2 cell-surface antigen heavy chain	-0.44	2.02×10^{-3}
Adipoq	Adiponectin	-0.88	2.18×10^{-3}
Dnpep	Aspartyl aminopeptidase	-0.51	3.21×10^{-3}
F13b	Coagulation factor XIII B chain	-0.34	4.66×10^{-2}
Col1a1	Collagen alpha-1(I) chain	-0.45	1.37×10^{-2}
Col1a2	Collagen alpha-2(I) chain	-0.37	3.43×10^{-2}
Ckm	Creatine kinase M-type	-1.45	2.71×10^{-2}
Cycs	Cytochrome c, somatic	-0.29	2.57×10^{-2}
Lap3	Cytosol aminopeptidase	-0.32	2.46×10^{-2}
Sod3	Extracellular superoxide dismutase [Cu-Zn]	-0.39	1.37×10^{-2}
Fabp3	Fatty acid-binding protein, heart	-2.34	1.07×10^{-2}
Hbq1b; Hbq1a	Globin d1; Globin d2	-0.93	4.41×10^{-2}
Gpi	Glucose-6-phosphate isomerase	-0.59	4.81×10^{-2}
Gpx1	Glutathione peroxidase 1	-0.5	3.38×10^{-2}
Gda	Guanine deaminase	-0.48	4.40×10^{-2}
	Ig heavy chain V region 441	-0.91	1.22×10^{-2}
	Ig kappa chain V-VI region XRPC 44	-0.73	1.87×10^{-2}
Ighv11-1; Ighv11-2	Immunoglobulin heavy variable 11-1 (Fragment); Immunoglobulin heavy variable V11-2 (Fragment)	-0.67	4.38×10^{-2}
Ighv13-2	Immunoglobulin heavy variable 13-2	-0.96	1.61×10^{-2}
Ighv2-5	Immunoglobulin heavy variable 2-5	-1.3	3.65×10^{-2}

Ighv5-6	Immunoglobulin heavy variable 5-6 (Fragment)	-1.37	4.25×10^{-2}
Ighv7-4	Immunoglobulin heavy variable 7-4	-1.42	9.60×10^{-4}
Ighv1-67	Immunoglobulin heavy variable V1-67	-1.04	1.54×10^{-2}
Ighv1-9	Immunoglobulin heavy variable V1-9	-1.23	3.39×10^{-2}
Igkv9-124	Immunoglobulin kappa chain variable 9-124	-0.65	3.86×10^{-2}
Igkv12-98	Immunoglobulin kappa variable 12-98	-1.1	1.08×10^{-2}
Iglc1	Immunoglobulin lambda constant 1 (Fragment); Ig lambda-1 chain C region	-0.78	2.73×10^{-2}
Iglv1	Immunoglobulin lambda variable 1 (Fragment); Ig lambda-1 chain V region; Ig lambda-1 chain V region S43	-0.57	2.65×10^{-2}
Il1r2	Interleukin-1 receptor type 2	-0.8	3.55×10^{-2}
Man2b1	Lysosomal alpha-mannosidase	-0.63	1.19×10^{-3}
Mdh1	Malate dehydrogenase, cytoplasmic	-0.96	3.41×10^{-2}
Mgam	Maltase-glucoamylase	-0.7	1.66×10^{-3}
Mb	Myoglobin	-1.96	2.18×10^{-4}
Me1	NADP-dependent malic enzyme	-0.73	1.91×10^{-3}
Ncam1	Neural cell adhesion molecule 1	-0.33	4.89×10^{-2}
Fap	Prolyl endopeptidase FAP	-0.53	6.15×10^{-4}
Psma2	Proteasome subunit alpha type-2	-0.19	4.18×10^{-2}
Psmb3	Proteasome subunit beta type-3	-0.26	1.48×10^{-2}
Psmb6	Proteasome subunit beta type-6	-0.55	0
Rdx	Radixin	-0.56	3.40×10^{-2}
Serpina3c	Serine protease inhibitor A3C	-0.71	8.45×10^{-4}
Vcp	Transitional endoplasmic reticulum ATPase	-0.79	3.84×10^{-2}
Umod	Uromodulin	-1.06	3.92×10^{-3}
Pepd	Xaa-Pro dipeptidase	-0.41	1.05×10^{-2}

Supplementary Table 4. Serum proteomics hits in the KPC mice. Protein hits identified in the serum proteomics screen, in the KPC (*Kras*$^{G12D/+}$ *Trp53*$^{R172H/+}$ *Pdx1-Cre*) mice at 12-weeks (+/- 10 days) old. 139 significantly increased hits and 44 significantly decreased hits. The gene name, protein name, log2(fold-change) and adjusted p-values for each hit are shown.

Gene symbol	Increasing Protein Hit	Log2(fold-change) (between PDAC patients and controls)	Adjusted p-value
C2	Complement C2	0.24	1.56×10^{-2}
C5	Complement C5	0.18	7.33×10^{-3}
C7	Complement component C7	0.29	2.02×10^{-2}
CFB	Complement factor B	0.28	8.00×10^{-3}
CFH	Complement factor H	0.17	1.86×10^{-2}
ITIH3	Inter-alpha-trypsin inhibitor heavy chain H3	0.44	2.02×10^{-2}
CD14	Monocyte differentiation antigen CD14	0.26	2.28×10^{-2}
PIGR	Polymeric immunoglobulin receptor	1.35	4.00×10^{-3}

VWF	von Willebrand factor	0.64	6.29×10^{-3}

Gene symbol	Decreasing Protein Hit	Log2(fold-change) (between PDAC patients and controls)	Adjusted p-value
ALB	Albumin	-0.21	3.52×10^{-2}
AGT	Angiotensinogen	-0.28	1.87×10^{-2}
APOA1	Apolipoprotein A-I	-0.67	1.29×10^{-2}
APOA2	Apolipoprotein A-II (Fragment)	-0.76	5.50×10^{-3}
APOC3	Apolipoprotein C-III	-0.51	1.78×10^{-2}
APOL1	Apolipoprotein L1	-0.56	1.67×10^{-2}
CNDP1	Beta-Ala-His dipeptidase	-0.58	3.44×10^{-2}
CRTAC1	Cartilage acidic protein 1	-0.50	4.58×10^{-2}
GSN	Gelsolin	-0.44	4.00×10^{-3}
HRG	Histidine-rich glycoprotein	-0.31	4.56×10^{-2}
IGFBP3	Insulin-like growth factor-binding protein 3	-0.47	2.40×10^{-2}
FN1	Isoform 1 of Fibronectin	-0.39	3.81×10^{-2}
LCAT	Phosphatidylcholine-sterol acyltransferase	-0.46	2.38×10^{-2}
PON1	Serum paraoxonase/arylesterase 1	-0.52	8.00×10^{-3}
CLEC3B	Tetranectin	-0.39	2.14×10^{-2}
TTR	Transthyretin	-0.49	9.33×10^{-3}

Supplementary Table 5. Serum proteomics hits in the early-stage PDAC patients. Protein hits identified in the serum proteomics screen in early-stage (I-II) pancreatic ductal adenocarcinoma (PDAC) patients, compared to benign symptomatic pancreaticobiliary disease case controls. 9 significantly increased hits and 16 significantly decreased hits. The gene name, protein name, log2(fold-change) and adjusted p-values for each hit are shown.

Supplementary Figure Legends

Supplementary Figure 1. PanIN GEMMs histology, AB/PAS staining and PCA plot. A– Representative pancreata histology images of haematoxylin and eosin (H&E) and Alcian blue (AB) /Periodic Acid-Schiff (PAS) of wild-type mice, *Pdx1-Cre*-only mice and *Pdx1-Cre Kras*$^{G12D/+}$ mice from each ATG colony (n=20 for each genotype and colony), as well as both impaired pancreatic autophagy genetically engineered mouse models (GEMMs) (*Pdx1-Cre Atg7*$^{fl/fl}$, n=19, and *Pdx1-Cre Atg5*$^{fl/fl}$, n=20) at 90-days (+/- 10 days) old. Images at 10X magnification with 500 µm scale bars shown. The wild-type H&E and Alcian blue/PAS images are also used as reference in Figure 1-B and Figure 2-B, as they are the same reference samples. **B**– Alcian Blue/PAS-positive staining was quantified in the pancreas tissue of male and female WT, ATG7 pancreatic intraepithelial neoplasia (PanIN) GEMM or ATG5 PanIN GEMM mice (n=5 per genotype). Data in graphs are presented as mean ± standard deviation. Statistical significance was analysed using the unpaired Student's t-test (GraphPad Prism 10.2.2). **p<0.01; ***p<0.001. **C**– Principal component analysis (PCA) plot of the 159 serum samples in the PanIN GEMM proteomics screen: 10 males and 10 females of each genotype in each ATG colony (except 9 males for ATG7 impaired pancreatic autophagy group). Each data point is one serum sample. Data points are coloured based on genotype: Wild-type (blue), Cre-only (yellow), Impaired Pancreatic Autophagy (orange) and PanIN (green) mice. Data points are shaped based on colony: ATG7 (triangles) and ATG5 (circles). Data are plotted based on the top two principal components, PC1 and PC2 respectively.

Supplementary Figure 2. KPC mouse serum proteomics PCA plot and Ki67 staining. A– Principal component analysis (PCA) plot of 37 serum proteomics samples: 18 wild-type (10 males and 8 females) and 19 KPC (*Kras*$^{G12D/+}$ *Trp53*$^{R172H/+}$ *Pdx1-Cre*) (9 males and 10 females) samples. Each data

point is one serum sample. Data points are coloured based on genotype: wild-type (light blue) and KPC (dark blue). Data points are shaped based on sex: male (triangles) and female (circles). Data are plotted based on the top two principal components, PC1 and PC2 respectively. **B**– Ki67-positive nuclei were quantified in the pancreas tissue of male and female wild-type (WT), ATG7 pancreatic epithelial neoplasia (PanIN) genetically engineered mouse model (GEMM), ATG5 PanIN GEMM or KPC mice (n=3 per genotype). Data in graphs are presented as mean ± standard deviation.

Supplementary Figure 3. Early-stage human PDAC patient serum proteomics PCA plot, heatmap of a publicly available dataset of human PDAC proteomics and serum CA19-9 levels. A– Principal component analysis (PCA) plot of 93 serum proteomics samples: 62 benign symptomatic pancreaticobiliary disease cases (controls) (26 males and 36 females) and 31 early-stage pancreatic ductal adenocarcinoma (PDAC) patients (15 males and 16 females). Each data point is one serum sample. Data points are coloured based on sample group: controls (light blue) and early-stage PDAC (dark blue). Data points are shaped based on sex: male (triangles) and female (circles). Data are plotted based on the top two principal components, PC1 and PC2 respectively. **B**– Heatmap of 4 of the protein hits (CFH, C5, ITIH3 and CD14) that were available in a publicly available proteomics dataset of human PDAC tissue (Tian *et al*, 2019, PMC6765243 – supplementary data 1)[1]. Gene symbols are shown on the right-hand side of the heatmap) (z-score blue (-2) to red (2), across 17 tissue samples: 2 normal, 2 PanIN, 2 pancreatitis, 3 well-differentiated PDAC, 4 moderately-differentiated PDAC and 4 poorly-differentiated PDAC). **C**– Serum levels of CA19-9 in 50 control cases (benign pancreaticobiliary disease cases) and 27 early-stage PDAC cases. Statistical significance was analysed using the unpaired Student's t-test (GraphPad Prism 10.2.2). $^{**}p<0.01$.

Supplementary Figure 4. Immunohistochemistry (IHC) staining of pre-PDAC KPC pancreas tissue for the hits ITIH3, CD14, CFB and CFH, as well as for α-SMA and vimentin as cancer-associated fibroblast (CAF) markers. Representative images of serial sections of pancreata of wild-type (WT, n=6) and pre-pancreatic ductal adenocarcinoma (PDAC) KPC ($Kras^{G12D/+}$ $Trp53^{R172H/+}$ *Pdx1-Cre*, n=6) mice stained for haematoxylin and eosin (H&E) and Alcian blue (AB)/Periodic Acid-Schiff (PAS), CD14, CFB, ITIH3, CFH, α-smooth muscle actin (α-SMA) and vimentin. Scale bars: 100 µm. Two sets of serial sections from the same tissue blocks were obtained and stained for either H&E, alcian blue/PAS, CD14, CFB, α-SMA and vimentin (set 1); or H&E, alcian blue/PAS, ITIH3, CFH, α-SMA and vimentin (set 2) to be consistent and to compare the stains.

Supplementary Figure 5. Additional Receiver Operator Characteristic (ROC) curves of pairwise and multiple protein combinations of hits from the early-stage PDAC patient serum proteomics screen data, on MetaboAnalyst. ROC curves were generated using the early-stage pancreatic ductal adenocarcinoma (PDAC) patient serum proteomics screen data (plotting 1-sensitivity against sensitivity), for: **A**– C5 and CFB, **B**– C5 and CFH, **C**– C5 and ITIH3, **D**– C5 and CD14, **E**– CFB and CFH, **F**– CFB and ITIH3, **G**– CFB and CD14, **H**– CFH and ITIH3, **I**– CFH and CD14, **J**– ITIH3 and CD14, **K**– C5, CFH, CD14 and CFB, **L**– C5, CFH, CD14 and ITIH3 and **M**– all 5 proteins combined (C5, CFH, CD14, CFB and ITIH3). Using the serum proteomics data of 62 benign symptomatic pancreaticobiliary disease case controls and 31 early-stage PDAC patient samples. Area under the curve (AUC) values and 95% confidence intervals (CIs) are shown on their respective ROC curves.

Supplementary Figure 6. Correlation analysis of gene expression of the 5 hits with the drug sensitivity to the KRAS inhibitor sotorasib in DepMap database. Correlation graphs between drug sensitivity for sotorasib and the gene expression of **A**– ITIH3, **B**– C5, **C**– CFB, **D**– CFH and **E**– CD14 obtained from the DepMap database. Gene expression of KRAS is also represented by the different colours of the data points (see legend in the figure). Pearson correlation coefficient and p values are shown on their respective correlation graphs.

References:

1. Tian C, Clauser KR, Öhlund D, Rickelt S, Huang Y, Gupta M, et al. Proteomic analyses of ECM during pancreatic ductal adenocarcinoma progression reveal different contributions by tumor and stromal cells. Proc Natl Acad Sci. 2019;116(39):19609-18.
